# Signatures of selection in recently domesticated macadamia

**DOI:** 10.1038/s41467-021-27937-7

**Published:** 2022-01-11

**Authors:** Jishan Lin, Wenping Zhang, Xingtan Zhang, Xiaokai Ma, Shengcheng Zhang, Shuai Chen, Yibin Wang, Haifeng Jia, Zhenyang Liao, Jing Lin, Mengting Zhu, Xiuming Xu, Mingxing Cai, Hui Zeng, Jifeng Wan, Weihai Yang, Tracie Matsumoto, Craig Hardner, Catherine J. Nock, Ray Ming

**Affiliations:** 1 Center for Genomics and Biotechnology, Fujian Provincial Key Laboratory of Haixia Applied Plant Systems Biology, Key Laboratory of Genetics, Fujian Agriculture and Forestry University, 350002 Fuzhou, China; 2 South Subtropical Crop Research Institute, China Academy of Tropical Agricultural Sciences, 524091 Zhanjiang, China; 3 USDA-ARS, Pacific Basin Agricultural Research Center, Hilo, HI 96720 USA; 4 Queensland Alliance for Agriculture and Food Innovation, The University of Queensland, St Lucia, QLD Australia; 5 Southern Cross Plant Science, Southern Cross University, Lismore, NSW Australia; 6 Department of Plant Biology, University of Illinois at Urbana-Champaign, Urbana, IL 6180 USA

**Keywords:** Genetic variation, Plant genetics, Agricultural genetics, Evolutionary genetics

## Abstract

Macadamia is a high value nut crop that is recently domesticated, ideal for testing the effect of artificial selection. Here, we sequence the genome of Hawaiian cultivar ‘Kau’ and assemble into 794 Mb in 14 pseudo-chromosomes with 37,728 genes. Genome analysis reveals a whole-genome duplication event, occurred 46.8 million years ago. Gene expansions occurred in gene families involves in fatty acid biosynthesis. Gene duplication of MADS-Box transcription factors in proanthocyanidin biosynthesis are relevant for seed coat development. Genome re-sequencing of 112 accessions reveals the origin of Hawaiian cultivars from Mount Bauple in southeast Queensland in Australia. Selective sweeps are detected in macadamia cultivars, including genes involved in fatty acid biosynthesis, seed coat development, and heat stress response. Such strong effects of artificial selection in few generations reveals the genomic basis for ‘one-step operation’ for clonal crop domestication. The knowledge gained could accelerate domestication of new crops from wild species.

## Abstract (web summary)

Macadamia is a recently domesticated nut crop. Here, the authors report the genome assembly of Hawaiian cultivar ‘Kau’ and conduct population genomic analyses to reveal the origin of Hawaiian cultivars and the genomic basis for one-step operation for the clonal crop domestication.

## Introduction

Plant domestication began ~12,000 years ago in the Fertile Crescent and played an important role in facilitating the rise of civilization^1,2^. Domestication is a complex evolutionary process, through which cultivars are selected that differ from their wild progenitors in quality, yield, or adaptation^3^. The evolutionary trajectory of this process from wild plants to cultivars is generally comprised of four stages^4^. Stage 1 is characterized by pre-domestication and the onset of domestication. In stage 2, frequencies of desirable alleles are increased through in situ selection of desirable germplasm. Formation of cultivated populations that are adapted to new environments and local preferences occurs in stage 3, and deliberate breeding of crop varieties to maximize yield, ease of farming, uniformity, and quality occur during stage 4. Most food crops have progressed to the late stages of domestication, and their genome structure and population diversity have been studied across many crops, most of these are at stage 3 or 4, and little is known about the impact of stages 1 and 2 on domestication.

Clonally propagated crops contribute substantially to agriculture and are a major part of the solution for global food security. However, crop improvement is often challenging with an unusual set of obstacles from male sterility, self-incompatibility, highly heterozygous genomes, and accumulation of deleterious mutations^5–7^. The ‘one-step operation’ hypothesis for the domestication of clonally propagated crops proposed that clonal crops often underwent only a few recombination-and-selection cycles followed by long-lasting clonal propagations^8^. This hypothesis was tested in self-incompatible pineapple cultivars^6^. Long tracks of terminal homology were detected in 10 chromosomes of the cultivar ‘Singapore Spanish’. These are likely the result of multiple mitotic recombination events at the single-cell stage of the clonal reproductive tissues, crowns, suckers, or slips selected for propagation and provide strong support for ‘one-step operation’ of domestication in this linage. Detectable mitotic recombination events are extremely rare, about 10^4^–10^5^-fold less frequent than meiotic recombination^9^. It is likely that thousands of years of clonal propagation would be required to reach terminal homology in 10 chromosomes^6^.

Macadamia (*Macadamia integrifolia* Maiden & Betche) is endemic to Australia and has a very short domestication history. The first orchards were established in Australia in the mid-1800s^10^ but it was in the 1930s that the crop was first commercialized in Hawaii^11,12^. Macadamia is unique as it has developed to be a global world tree nut crop after only 100 years of domestication^13^. Macadamia produces a premium kernel, that is encased in a very hard shell13 and contains the highest proportion of oil (up to 82%) of nut crops^14^. *Macadamia* is a member of the ancient Gondwanan family Proteaceae, comprised of four species (*M. integrifolia* and *M. tetraphylla* L.A.S. Johnson—which produce edible kernel, and *M. ternifolia* F. Mull and *M. jansenii* C.L. Gross & P.H. Weston that produce small inedible kernels high in cyanogenic glycosides). *Macadamia* is endemic to the sub-tropical rainforest of Eastern Australia and is one of the few crops domesticated from basal dicots^15^.

The first record of European cultivation of the plant is a tree planted in Brisbane, Australia, in 1857 following colonization of the region in the 1830s^15^. Initial commercial development of the crop occurred in Hawaii with seedlings from collections made from *M. integrifolia* grown from seed introduced into Hawaii in the late 19th century^11,13^. Following the development of reliable grafting technologies in the mid-1930s, selections were made from existing commercial seedling orchards in Hawaii for evaluation and release as cultivars for the establishment of clonal orchards. The first round of cultivars was released in the 1950s, with subsequent generations of evaluation of open-pollinated seedlings collected from the first-generation cultivars. Despite the global development of the industry from the 1970s, world production is still dominated by cultivars developed in Hawaii, including some first-generation cultivars. Evidence from chloroplast genome resequencing suggests that almost all of the Hawaiian germplasm traces back to a single small wild population in the north of the natural distribution of *M. integrifolia* south-west of the town of Gympie^13^. Nevertheless, genetic development of the crop has also occurred in other countries including Australia, South Africa, California, and China using Hawaiian germplasm and other wild samples including *M. tetraphylla* germplasm. Thus, commercial macadamia cultivars are only two–four generations from the wild. Important traits for domestication have been yield (albeit under weak genetic control), tree size, nut and kernel size, kernel recovery, and adaptation to warm or cooler environments^16^.

Macadamia is recognized as the world’s premium nut due to the high content and quality of oil and distinctive taste^13,17^. Edible macadamia kernels, mainly harvested from two species and their hybrids (*M. integrifolia*, *M. tetraphylla*) are cultivated in tropical and subtropical regions worldwide, including Australia, Hawaii, New Zealand, China, South Africa, South America, and Southeast Asia^15^. The two most noticeable features of macadamia are hard shells and high oil content, which motivated the domestication of this crop. Macadamia kernel is particularly rich in monounsaturated fat palmitoleic acid, which makes up 17% of its total oil content^14^. Palmitoleic acid is an omega-7 monounsaturated fatty acid with reported human health benefits including inflammation reduction and prevention of diabetes and cardiovascular diseases^18^, however, the evolutionary mechanisms of palmitoleic acid accumulation little are known. Seed coat plays a pivotal role in the protection of both the developing embryo and against deleterious biotic and abiotic influences before germination. Macadamia nutshell is well known for its surprisingly high strength, and 1800–4000 newton (N) are needed for breaking it^19,20^. However, the genetic mechanism underlying the development of seed coats in macadamia is unknown.

Macadamia has such a short domestication history with clear records and relatively intact natural populations. As such, this crop offers an exact beginning point of a ‘one-step operation’ of a clonally propagated crop and a rare opportunity to test the effect of intensive artificial selection in just a few generations. The first-generation *Macadamia integrifolia* Hawaiian cultivar HAES 344 (‘Kau’) was selected from the Nutridge seedling orchard in Hawaii in the 1930s^11^_._ Here, the genome of ‘Kau’ is sequenced along with 112 re-sequenced macadamia genomes, including 70 cultivars and selected lines and 42 wild accessions, to identify signatures of selection for important traits, domestication origins, and to understand the impact of early-stage selection on genome structure.

## Results

### Genome assembly and annotation

**Fig. 1:**
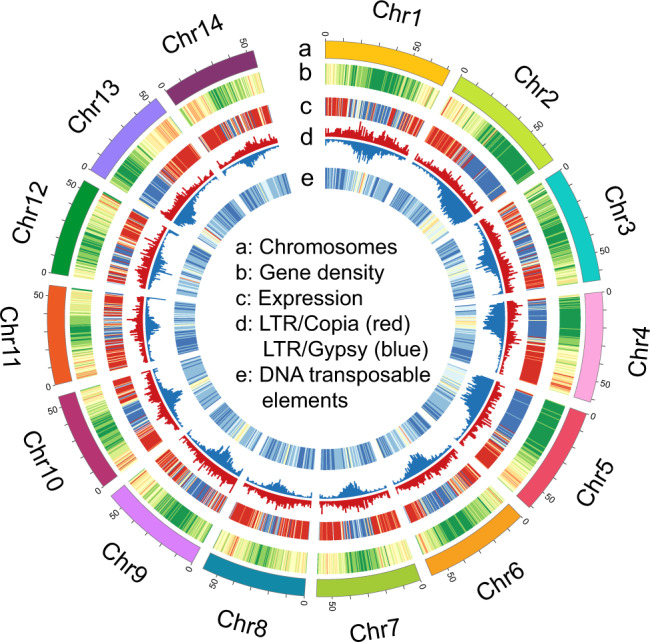
Distribution of genomic features along the macadamia genome. **a** karyotype in Mbp. **b** Gene density, red means high density, and green indicate low density. **c** Gene expression, red indicates high expression level, and blue means low. **d** LTRs distribution in chromosomes. **e** DNA transposable elements distribution in chromosomes, red means high density and blue means low density.

The genome size of ‘Kau’ was estimated to be 890 Mb by flow cytometry (Supplementary Table 1), consistent with the previous k-mer based estimate for macadamia of 896 Mb for ‘Mauka’^21^, a close relative of ‘Kau’^22^. We generated 89 Gb (100×) of long read from the PacBio Sequel II platform and 46 Gb (50×) of short-read sequence data from Illumina NovaSeq (Supplementary Table 2). The initial contig level assembly using CANU 1.7 yielded 1.10 Gb of assembled sequences, indicating that some heterozygous regions were assembled twice (Supplementary Table 3). To eliminate redundant sequences, Illumina reads were mapped to the assembled contigs to identify duplicated sequences, i.e. allelic haplotypes, resulting in the removal of 295 Mb sequences from the initial contig assembly. The assembled genome was 794 Mb, with a contig N50 of 281 kb (Supplementary Tables 3 and 4). Chromosomal level assembly of the ‘Kau’ genome was achieved using high-throughput chromatin conformation capture (Hi–C) for physical mapping to anchor scaffolds, resulting in 14 pseudo-chromosomes that anchored 794 Mb (99.97%) of the genome (Fig. 1 and Supplementary Fig. 1 and Supplementary Table 5).

BUSCO analysis of 1375 conserved single copy plant genes revealed 92.1% completeness with only 66 genes missing in the macadamia assembly (Supplementary Table 4). Alignment of RNA-seq assembled transcripts to the assembly showed 99.99% of base accuracy (Supplementary Table 6). In addition, 99.5% (165.23/166.03 million) of Illumina short reads were mapped to the assembly, covering 99.0% of the genome (Supplementary Table 7).

Genome annotation resulted in 37,728 protein-coding genes with 88.4% BUSCO completeness and 113 microRNAs (Supplementary Tables 4 and 5). In addition, we predicted 461.07 Mb of repetitive sequences, accounting for 57.0% of the assembled genome, including 49.0% retrotransposons and 8.8% DNA transposons (Supplementary Table 8). Long terminal repeat (LTR) retrotransposons were the major components, containing 266.9 Mb of sequences and accounting for 33.0% of the genome with 16.5 Gypsy and 6.4% Copia. The LINE retrotransposon content is unusually abundant and higher than that of Copia at 11.5% of the genome. A recent burst of Gypsy retrotransposons and an ancient burst of LINE elements were detected (Supplementary Fig. 2).

### Comparative genomic analysis

**Fig. 2:**
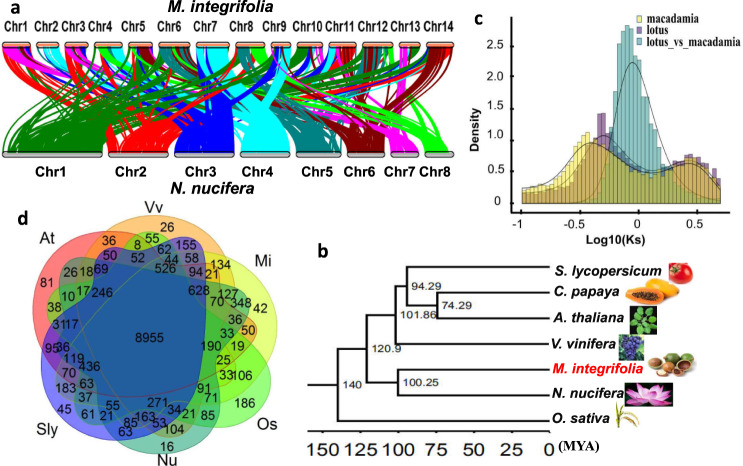
Evolutionary comparison and gene conservation of the macadamia (*M. integrifolia*) genome. **a** Inter-genomic comparison between *M. integrifolia* and *N. nucifera*. **b** Inferred phylogenetic tree across seven plant species including macadamia, calibrated using the divergence time of *A. thaliana* and *C. papaya* (68–72 million years ago) and monocot and eudicot (120–140 million years ago) as calibrators. **c** Synonymous substitution rate (*K*s) distributions of syntenic blocks for *M. integrifolia* and paralogs and orthologs with *N. nucifera* as shown by colored lines. **d** Shared gene families among At = *A. thaliana*, Os = *O. sativa*, Sly = *S. lycopersicum*, Vv = *V. vinifera*, Nu = *N. nucifera*, and Mi = *M. integrifolia*. The six species contain 8955 common gene families, and *M. integrifolia* has 42 specific gene families.

Comparative genomic analysis of macadamia *M. integrifolia* and lotus *Nelumbo nucifera* showed fragmented conserved synteny (Fig. 2a and Supplementary Fig. 3), and identified 1:1 syntenic depth ratios in the Macadamia-lotus and lotus-Macadamia, respectively. Macadamia chromosome 1 aligned with parts of lotus chromosome 2 and 7, whereas lotus chromosome 7 aligned with parts of macadamia chromosomes 1, 3, 10 11, and 13. In general, each macadamia chromosomes is aligned to parts of 2 or more of the 8 lotus chromosomes, and each lotus chromosome is aligned to parts of 4 or more of the 14 macadamia chromosomes. The close relationship of macadamia and sacred lotus is also confirmed in the maximum likelihood phylogeny of 898 gene orthologs (Fig. 2b). The divergence time between macadamia and lotus is estimated at 100.3 million years ago (MYA) (Fig. 2b), and a whole-genome duplication (WGD) in the macadamia lineage occurred about 42.3 MYA (*K*s = 0.35; Fig. 2c and Supplementary Fig. 4).

The analysis of gene families shared between ‘Kau’ and the representatives of six other species of diverse lineages including five eudicots and one monocot resulted in 213,308 proteins (67.43% of the input sequences) clustered into 14,999 groups (Supplementary Table 9) with 8955 gene families shared across the six lineages (Fig. 2d and Supplementary Table 9). Of 37,728 macadamia proteins, 26,889 clustered into 13,183 groups, of which 42 clusters were macadamia-specific and contained 222 proteins (Fig. 2d). These species-specific genes were distributed across all 14 macadamia chromosomes (Supplementary Fig. 5). KEGG pathway analysis identified many species-specific genes related to environmental adaptation (Supplementary Fig. 6). There were also 10,853 singleton proteins unique to macadamia (Supplementary Table 10).

### Macadamia shell development

**Fig. 3:**
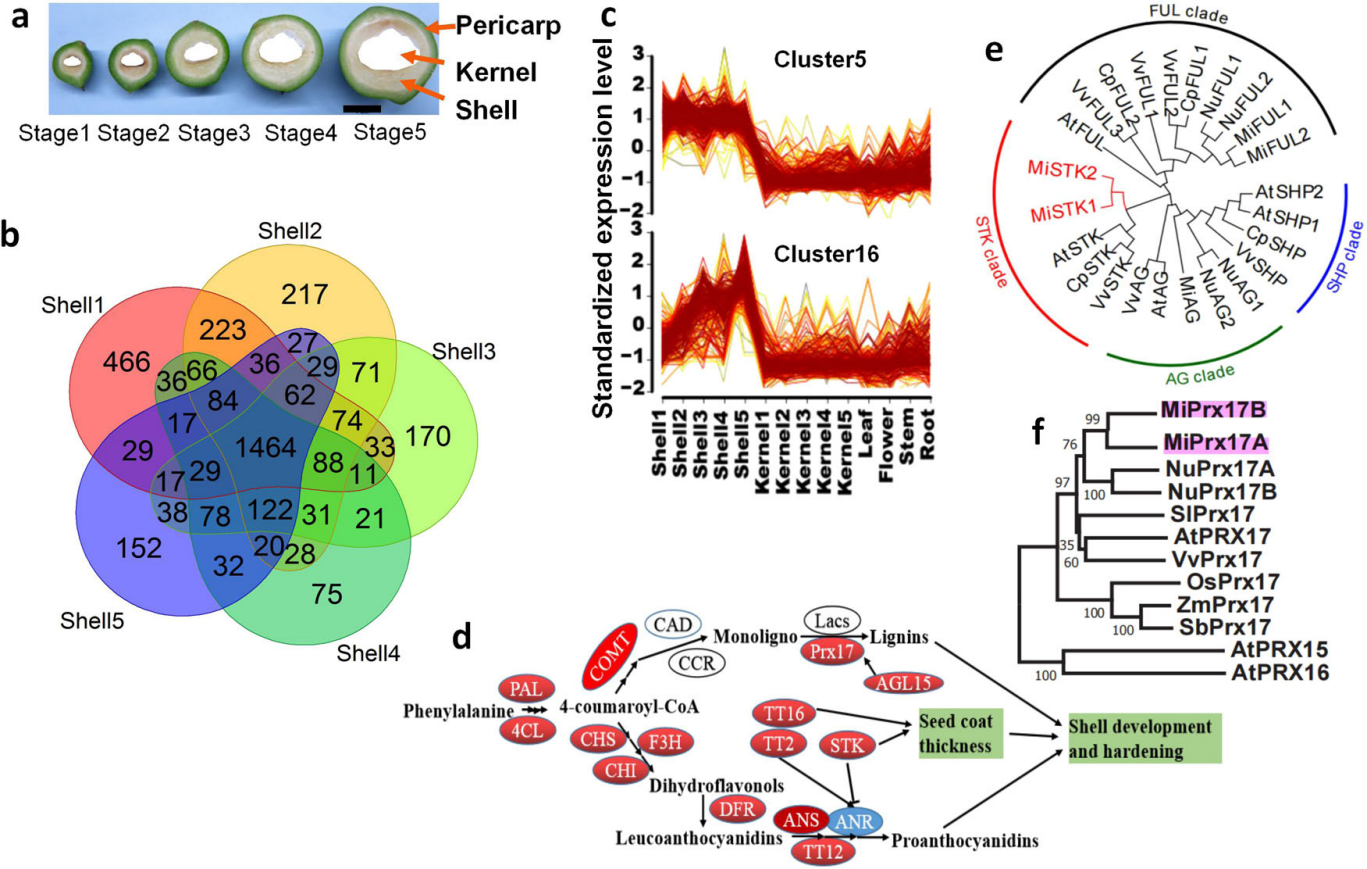
Shell development in *M. integrifolia*. **a** Phenotype of shell and kernel at different stages of fruit development. **b** Venn plot of up expression genes in stage Stage 1, Stage 2, Stage 3, Stage 4, and Stage 5 of shells. **c** Mfuzz clustering of differentially expressed transcripts in shell, kernel, and other tissues. **d** Schematic of shell development and hardening. A proposed model of *STK*, *TT16,* and *Prx17* in regulation of shell development and hardening. **e** Phylogenetic tree of *STK*, *AG*, *FUL,* and *SHP* genes in multiple species, including *M. integrifolia*, *N. nucifera*, *A. thaliana*, *V. vinifera,* and *C. papaya*. *STK*, *AG*, *FUL,* and *SHP* clades are indicated in different colors. **f** Phylogenetic tree of *Prx17* in *M. integrifolia*, *N. nucifera*, *A. thaliana*, *V. vinifera*, *S. lycopersicum*, *Z. mays* L., *O. sativa*, and *S. bicolor*.

There were 2735, 2641, 2337, 2201, and 2235 differentially expressed genes (DEGs) in five stages of formation of the macadamia shell (Fig. 3a) examined, with 1464 DEGs shared by all stages (Fig. 3b). Following grouping of 3845 DEGs correlated to shell development into 16 clusters based on their expression patterns (Fig.3c and Supplementary Fig. 7 and Supplementary Table 11), DEGs from clusters 5 and 16 showed high expression levels in shells compared to other tissues (Fig. 3c).

Expression of genes in cluster16 exhibited an upward trend that was highly expressed in the late stage of shell development (Supplementary Table 12). Most of these genes are involved in histogenesis and development of phloem or xylem, secondary cell wall formation, and lignin biosynthesis (Supplementary Table 13). In contrast, genes in cluster5 are highly expressed in every developmental stage of shells (Supplementary Table 14). Many are key transcription factors of organ formation and morphogenesis, essential genes in pathway of phenylpropanoid or flavonoid biosynthesis, or sugar transporters (Supplementary Table 15).

Notably, *SEEDSTICK* (*STK*) and *TRANSPARENT TESTA 16* (*TT16*), which encodes a MADS-domain transcription factor as a master regulator of development and metabolism of the seed coat, are highly expressed in shells (Supplementary Figs. 8, 9). Two paralogs of *STK* (*MiSTK1* and *MiSTK2*) were identified in the macadamia genome in Chr8 and Chr6, respectively, resulting from duplications. We also identified one *STK* orthologs in *Arabidopsis thaliana*, *Vitis vinifera,* and *Carica papaya* genomes, but no copy in *N. nucifera* (Fig. 3e).

Macadamia *MiSTK1*, *MiSTK2* are highly similar in coding sequences, protein sequences, and gene structure (Supplementary Figs. 10, 11). We found that all three *MiSTKs* showed very similar expression patterns in flowers, leaves, stems and roots, but were strongly expressed in shells (Supplementary Fig. 8).

We identified orthologs of the class III peroxidase *PRX17*, which regulate age-dependent lignified tissue formation, in the macadamia and seven other genomes with two paralogs in macadamia, two paralogs in lotus, and one ortholog in each of other six species (Fig. 3f). The two paralogs of macadamia *MiPRX17* (*MiPRX17A* and *MiPRX17B*) are highly similar in coding sequences, protein sequences, and gene structure (Supplementary Fig. 10c, d). While *MiPRX17A* and *MiPRX17B* showed very similar expression patterns in flowers, leaves, stems, and roots, they were strongly expressed in shells (Supplementary Fig. 8). We also detected strong expression of *MiAGL15* in macadamia shell, an ortholog of *AtAGL15* that directly regulates *AtPRX17* by directing binding to the CARGCW8 cis-element^23^. Promoter analysis showed that *MiPRX17A* has one putative binding site for *MiAGL15* and *MiPRX17B* has one *cis*-element related to lignin biosynthesis (Supplementary Table 16).

Accumulation of proanthocyanidins (PAs) in the innermost layer of the seed coat is necessary for functional seed coat growth and also a characteristic feature of seed coat development^24,25^. Among the high expression genes in shells, many genes are related to PAs biosynthesis pathway, including *PALs*, *4CLs*, *COMTs*, *CHSs*, *CHIs*, *F3H*, *DFRs*, and *TT12* (Supplementary Fig. 9).

### Fatty acid biosynthesis in macadamia kernel development

**Fig. 4:**
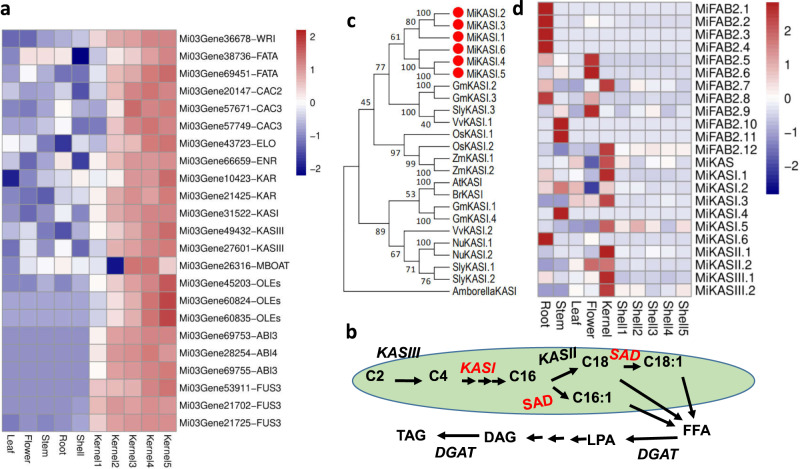
Kernel development in *M. integrifolia*. **a** Expression of fatty acid biosynthesis-related genes in kernels and other tissues of macadamia. **b** Schematic of lipid biosynthesis pathway. KAS, ketoacyl synthases; SAD stearoyl-ACP desaturase, DGAT diacylglycerol acyltransferase. **c** Phylogeny of the *KASI* genes family in *M. integrifolia*. LPA lysophosphatidic acid, DAG diacylglycerol, TAG triacylglycerol, FFA free fatty acid. **d** Expression of fatty acid biosynthesis genes in tissues of *M. integrifolia*. Source data underlying **a**, **d** are provided as a Source Data file.

There were 1953, 1610, and 1417 DEGs in the Kernel 1 (Stage 1), Kernel 3 (Stage 3), and Kernel 5 (Stage 5) three stages of kernel development, with 579 DEGs shared by three stages (Supplementary Fig. 12a). All these DEGs were filtered from kernel samples for weighted gene co-expression network analysis (WGCNA). Cluster analyses of the DEGs indicated a higher correlation between similar tissues/developmental stages. Stage 1 kernel transcriptomes clustered separately to all others while those of the later three development stages clustered together and showed substantial differences to those of other tissues (Supplementary Fig. 12b). Module–trait relationships analysis shows that the blue and gray module is highly related to fatty acid biosynthesis during kernel development (Supplementary Fig. 13a). There are 591 and 488 genes in the blue and gray module individually that are highly correlated with kernel development (Supplementary Fig. 13b). Kyoto Encyclopedia of Genes and Genomes (KEGG) pathway analysis of these highly correlated genes showed they were significantly enriched in lipid metabolism (Supplementary Fig. 14), and included orthologs of known seed oil biosynthesis control transcription factors, *Wrinkled1* (*WRI*), *Abscisic Acid3* (*ABI3*) and *Fusca3* (*FUS3*) (Fig. 4a). Other genes that are involved in fatty acid biosynthesis and oil assembly are also highly expressed in kernel tissues, including *Fatty Acyl-acp Thioesterases A* (*FATA)*, *Biotin Carboxylase* (*CAC), Enoyl-acp Reductase (ENR)*, *3-Ketoacyl-CoA Reductase (KAR)*, *3-Ketoacyl-CoA Synthase (KAS)*, *Elo homolog 2 (ELO2)*, *Membrane-Bound O-acyl Transferase (MBOAT)* and *Oleosin (OLE)* (Fig. 4a).

In the macadamia genome, 269 clusters (3955 genes) were substantially expanded, with 16 (1704 genes) contracted compared with other plant genomes in an analysis undertaken to investigate the genomic basis of selected metabolite biosynthesis (Supplementary Table 18). KEGG pathway analysis of the expanded genes revealed marked enrichment in functions related to fatty acid metabolisms, such as fatty acid biosynthesis, elongation and degradation, palmitate biosynthesis, stearate biosynthesis, *cis*-vaccenate biosynthesis and cutin, suberine, and wax biosynthesis. (*p*-value < 0.05, Supplementary Fig. 15, Supplementary Table 19). We identified gene families related to fatty acid chains elongation (Fig. 4b), desaturation, and acyl transfer such as ketoacyl synthases (*KAS*), stearoyl-ACP desaturase (*SAD*), fatty acid desaturases (*FAD*), diacylglycerol acyltransferase (*DGAT*), and acyl-CoA:sn-glycerol-3-phosphate acyltransferase (*GPAT*) in 14 species (Supplementary Table 20). Comparison of gene numbers in macadamia to other species showed distinct gene family expansions of the *KASI* and *SAD* genes. For *KASI* in particular there were six paralogs in macadamia, and one ortholog each in *A. thaliana*, *B. rapa*, *P. dulcis*, and *A. duranensis* (Fig. 4c). *KASI* is responsible for the elongation of fatty acid chains from enoyl-ACP (4:0-ACP) to palmitoyl-ACP (16:0-ACP).

Phylogenetic analysis of *KASI* and *FAB2* proteins from macadamia and other species showed that the six copies of *KASI* and *FAB2* in macadamia had a very close relationship (Fig. 4a and Supplementary Fig. 12c), although the genomic regions do not share synteny. *MiKASI1*, *MiKASI3* and *MiKASI5 MiFAB2.7* and *MiFAB2.12*, in particular, exhibited dramatically higher expression in kernels (Fig. 4d).

### Genetic diversity and domestication origin

To explore genetic diversity and the brief domestication history, 112 macadamia accessions were re-sequenced, including 59 cultivars and selected lines, and 42 wild accessions, seven hybrid cultivars, and four outgroup species (Supplementary Table 20). *Macadamia integrifolia* is distributed over ~250 km in lowland subtropical rainforest fragments of eastern Australia. To identify the origins of domestication, wild accessions were sourced primarily from three population clusters (C1–C3) north of Brisbane in Queensland. Evidence from previous genetic studies indicates that the Hawaiian cultivars originated from the northern range of *M. integrifolia*. All *M. integrifolia* individuals formed a clade distinct from *M. tetraphylla* and hybrids (Supplementary Fig. 16a). This was further supported by a principal component analysis (PCA) (Supplementary Fig. 16b), population structure, and linkage disequilibrium (LD) analysis (Supplementary Fig. 16c). Structure analysis identified two population clusters (*K* = 2) that clearly separate *M. integrifolia* and *M. tetraphylla* accessions. Three population clusters (*K* = 3) clearly distinguish *M. integrifolia* cultivars from wild individuals (Supplementary Fig. 17a, b). Wild *M. integrifolia* accessions were assigned to three main regional groups (C1–C3) at *K* = 4 (Supplementary Fig. 17a).

**Fig. 5:**
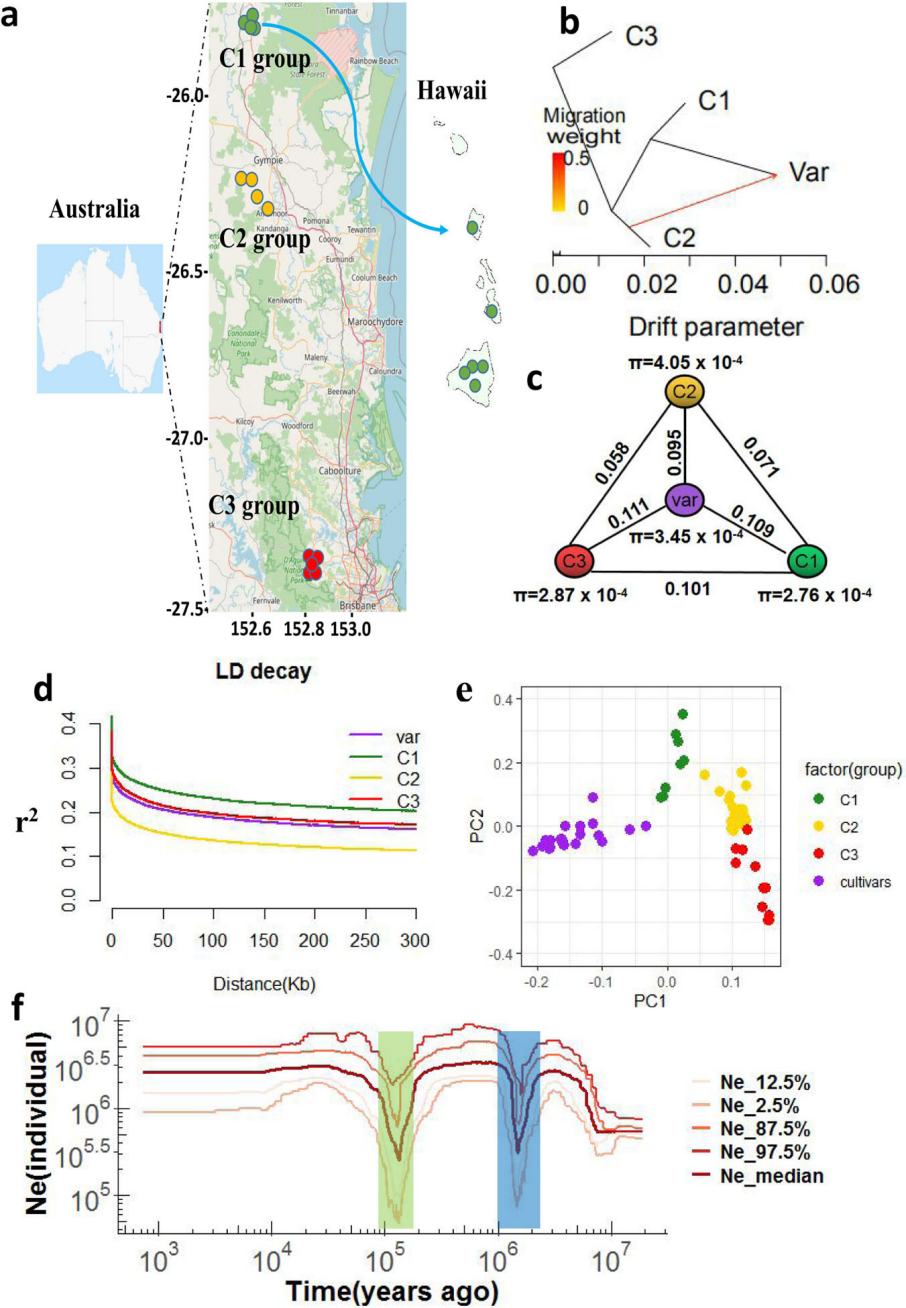
Geographic distribution and drift paths of wild to varieties. **a** Genetic-flow paths visualized on the map, the map was based on OpenStreetMap (Base map © OpenStreetMap, see https://www.openstreetmap.org/copyright) **b** Gene-drift in wild groups and varieties. **c** *F*_ST_ and *π* values of each groups. **d** LD decay for three wild groups (C1–C3) and varieties of *M. integrifolia*. **e** PCA clustering of three wild groups (C1–C3) and varieties of *M. integrifolia*. **f** Effective population size (*N*_e_) history estimated using ANGSD using *g* (generation time) = 8 years and *m* (neutral mutation rate per generation) = 4.175*10^−9^ and plot by software Stairway plots with 200 bootstrap iterations. Source data underlying **d**, **f** are provided as a Source Data file.

PCA analysis was carried out using 25 Hawaiian cultivars and 35 wild accessions to assess genetic relationships among Hawaiian cultivars and three wild groups. These 60 accessions were classified into four geographic groups, C1–C3 and Hawaii cultivars, (Fig. 5 and Supplementary Table 22). LD decay shows wild group C2 has a fast decay rate and followed by Hawaiian cultivars, C3 and C1 group (Fig. 5d). Fixation index (*F*_ST_) was calculated among Hawaiian cultivars and three wild groups. Genetic differentiation between Hawaiian cultivars and the C3 group (*F*_ST_ = 0.111) was the largest, between Hawaiian cultivars and the C2 group (*F*_ST_ = 0.095) the smallest, and between cultivars and the C1 (*F*_ST_ = 0.109) intermediate, (Fig. 5c). The nucleotide diversity of each group was estimated. Group C2 had the highest average nucleotide diversity (*π*) of 4.05 × 10^−4^, followed by Hawaiian cultivars (3.45 × 10^−4^), C3 group (2.87 × 10^−4^), and C1 group (2.76 × 10^−4^) (Fig. 5C). Nucleotide diversity of the Hawaiian cultivars was most similar to that of the C2 group. The most northerly wild *M. integrifolia* populations are located in the Mt Bauple (C1) and Gympie (C2) regions of southeast Queensland that are separated by over 70 km (Fig. 5a). The chloroplast genome phylogeny (Supplementary Fig. 17b) is concordant with previous evidence that the maternal lineage of commercial cultivars developed in Hawaii originated in the Gympie region^15^. The nuclear SNP phylogeny, however, provides strong and conflicting support for an Mt Bauple (C1) origin of domestication (Supplementary Fig. 17c). The nuclear C1 clade also includes the Hawaiian cultivars suggesting that the most recent common ancestor of the cultivars was from Mt. Bauple. Individuals from each of the three sampled geographic regions (C1–C3) form deeply divergent monophyletic clades in both nuclear and chloroplast phylogenies. One exception is a C1 individual with a C2 chloroplast haplotype (W04-MB04) and 50% admixed ancestry from the two regions (Supplementary Fig. 17a) suggesting introgression following seed dispersal from Gympie to Mt. Bauple. Other C1 individuals are admixed with lower levels of C2 ancestry. Translocation of seed between regions was presumably human-mediated given that small rodents, gravity and water are the proposed mechanisms for natural seed dispersal^13^. Phylogenetic network analysis in Treemix indicates that the Hawaiian cultivars are derived from the C1 lineage with C2 ancestry (Fig. 5b).

### Signatures of selection at early stage of domestication

Historical effective population size (*N*_e_) analyses indicated that *M. integrifolia* population size has remained stable over the past 100,000 years ago but underwent two historical Ne declines. The most recent decline was ~200,000–100,000 years ago and an earlier decline at 1,700,000–1,100,000 years ago (Fig. 5f).

**Fig. 6:**
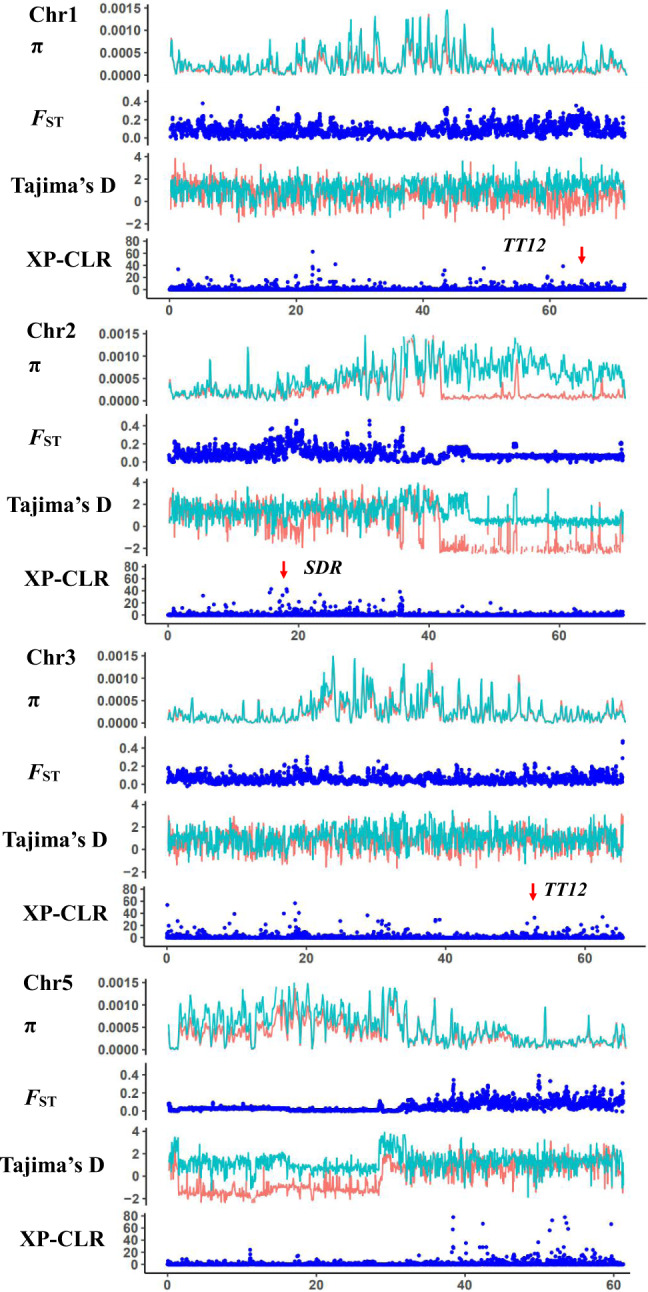
Signatures of selected signals in chromosomes 1, 2, 3, and 5 of *M. integrifolia* genome. The upmost dotplot is nucleotide diversity (*π*) values, red indicate varieties and green line indicate wild group; the second lay is fixation index (*F*_ST_) between the wild and cultivated macadamia accessions; the third lay is Tajima’s *D* values, red indicate varieties and green line indicate a wild group. The bottom is the genome-wide distribution of selective-sweep signals identified based on the cross-population composite likelihood ratio test (XP-CLR). *TT12*, TRANSPARENT TESTA; SDR short-chain dehydrogenase reductase, ANS anthocyanidin synthase.

To screen for signatures of selection, *π*, *F*_ST,_ Tajima’s *D,* and XP-CLR were calculated across the genome of *M. integrifolia*. Low genetic diversity was detected by *π* and *F*_ST_ values of cultivars and wild accessions in a large portion of Chr2 and more than half the chromosome in Chr5 (Fig. 6 and Supplementary Figs. 18–27), which corresponded to the regions with unusually high content of Gypsy retrotransposons (Fig. 1d, blue color), resulting in a low rate of or no recombination. The signals from Tajima’s *D* in these two regions are artifacts, caused by very small starting plant materials selected from Mt. Bauple (C1), as no signals of selective sweeps were detected by XP-CLR in these regions.

Signals of artificial selection were detected in 126 blocks containing 284 protein-coding genes (Supplementary Fig. 28a). These had Tajima’s *D* values that were negative in cultivars and positive in wild accessions. These 284 genes represent 0.75% of the 37,723 genes available for selection.

Functional analysis revealed that the 284 genes under selection were enriched in several biological processes, with major groups including response to stimulus, metabolic process, single-organism process, and cellular process (Supplementary Fig. 29). KEGG enrichment identified multiple pathways related to biosynthesis or metabolism secondary metabolites that are involved in the response to biotic or abiotic stress, including flavonoid biosynthesis, monoterpenoid biosynthesis, diterpenoid biosynthesis, biosynthesis of secondary metabolites, stilbenoid, diarylheptanoid, and gingerol biosynthesis (Supplementary Fig. 30).

PAs are synthesized through the phenylpropanoid biosynthesis pathway and play an important role in seed development^24^. In particular, *ANS* and *TT12* play important roles in PAs biosynthesis. Analysis of the overlapping regions of XP-CLR, *F*_ST_, and Tajima’s *D* between macadamia wild populations and cultivars provide evidence for selection of *ANS* and *TT12* (Fig. 6 and Supplementary Fig. 28b). RNA-seq analysis showed that these genes were differentially expressed among the six tested tissues, with two *ANS* genes and *TT12* highly expressed in shells (Supplementary Fig. 9). In addition, there were signatures of selection for *Long-Chain Acyl-CoA Synthetase* (*LCAS*), a gene known for affecting storage oil synthesis and plant height^26–28^, and transcriptome analysis showed differential expression in different tissues (Supplementary Fig. 28).

Of the 284 selective genes, we identified three related to heat response (*HSFB4*, *THF,* and *HSF3*). RNA-seq analysis showed that these three genes were differentially expressed among six tested tissues, with high expression of one gene in flower and leaf, and one in the kernel, and one in the shell (Supplementary Fig. 28c).

Terminal runs of homozygosity^29^ are the hallmark of mitotic selection, so this is an effective genomic analysis method to distinguish sexual recombination from the ‘one-step operation’ for the domestication of clonally propagated crops. We analyzed terminal runs of homozygosity using SNPs from single-copy genes on each chromosome (Supplementary Fig. 31), however, no significant extensive terminal runs of homozygosity were identified in macadamia cultivars (Supplementary Figs. 32–45).

## Discussion

Most modern macadamia cultivars are just two to four generations from their wild ancestors, and the most widely cultivated Hawaiian cultivars are just two generations away^13^, which appeared to be in stage 1 of domestication^4^. The Hawaiian macadamia cultivars were selected from plant materials imported from Australia between 1882 and 1892, and the first large-scale commercial planting without selection as a crop started in 1922. A macadamia breeding program was initiated at Hawaii Agricultural Experiment Station in 1936. The first five cultivars were selected from 20,000 open-pollinated seedlings and released in 1948 and additional cultivars were released in subsequent years^30^. These Hawaiian cultivars and other cultivars selected in Australia using the same approach were propagated through grafting and are still in production. They are living examples of ‘one-step operation’ for domestication. Interspecific hybrid cultivars were selected in Australia in 1948^31^. Like pineapple, sexual recombination and ‘one-step operation’ co-exist among macadamia cultivars as interspecific and bi-parental hybridization were used in macadamia breeding programs, mostly in Australia, resulting in the release of several hybrid cultivars^31^. The lack of long terminal runs of homology in macadamia chromosomes is expected, which requires long-term clonal propagation in hundreds to thousands of years^6^, while only 2–4 generations of macadamia breeding were recorded over the past 100 years. With such a brief history of domestication, it is not a surprise that there was no evidence for fluctuation in effective population size during the past 100,000 years. The Ne decline estimated at ~200,000–100,000 years ago could be due to contraction of rainforest habitat during glacial maxima, and the other at 1,700,000–1,100,000 years ago could be caused by upheaval during the Calabrian Stage of the Pleistocene^32^.

Selective sweeps were detected in many chromosomes of Hawaiian cultivars, even though the intensive selection of certain traits has only been applied over two generations. This is unexpected because selective sweeps were usually detected in crops that have been domesticated thousands of years ago over thousands of generations, and further validate the hypothesis of ‘one-step operation’ in clonally propagated crops as reported in pineapple^8^. Macadamia cultivars and genomic technologies that enabled large-scale genome re-sequencing offered a rare opportunity to examine the genomic basis of ‘one-step operation’. These detected selection signals in many chromosomes indicate that even just two recombination-and-selection cycles could yield genomic alteration favoring crop production for fruit quality and yield, which would persist in clonally propagated cultivars (Fig. 6). Among the 284 genes under selection, favorable alleles related to fatty acid biosynthesis, seed coat development, and heat stress response were selected by superior performance on yield and kernel quality of Hawaiian macadamia cultivars, fitting the definition of stages 2 of domestication, rather than stage 1 by just two generations separated from their wild ancestors^4^. This is even more impressive given modern breeding techniques of effective experimental design and stringent statistical analysis were not used in the development of the early Hawaiian cultivars^11^. This response to selection is therefore likely to be for traits with high individual heritability or for those selected for in 2nd stage testing where clpona replication was used to increase trait heritability, skipping the inefficient ‘unconscious’ selection in pre-historic time in the early stage of domestication of major crops. The selected genes and alleles in macadamia in two generations are likely the genomic basis for the success of ‘one-step operation’ for the domestication of clonally propagated crops^8^. The international isolation until the mid-19th century of the region of Australia where macadamia naturally occurs is the main reason for the apparently short domestication history of the plant, although it is unknown to what extent indigenous Australians modified native flora.

With the availability of a high-quality reference genome assembly and re-sequenced genomes of Hawaiian cultivars and wild accessions from south-east Queensland Australia, we have an unprecedented opportunity to clarify relationships between domesticated Hawaiian cultivars and wild germplasm, which is important to guide future macadamia breeding projects and prioritize conservation of wild germplasm. Population structure analysis suggests admixture in the most northerly wild *M. integrifolia* clade (C1) with the C2 and C3 clades (Supplementary Fig. 17a), indicating that C1 at Mt. Bauple may not represent a pure wild population. Migration phylogenetic tree, migration-drift, and principal component analyses all indicated that the C2 region and Mount Bauple (C1) from the northern distribution of the species in south-east Queensland, Australia is the original source of Hawaiian cultivars (Fig. 5). The C2 chloroplast clade included individuals from Mooloo and Mt. Bauple that shared a single chlorotype with many Hawaiian cultivars^15^. Chloroplast genomes have slower rates of molecular evolution due to uniparental inheritance, conserved genome structure, and gene content and play a vital role in photosynthesis among encoded genes^33^. Analyses from re-sequenced nuclear genomes yielded higher resolution to separate these three groups from this shared chlorotype. Re-sequencing of the chloroplast and nuclear genomes reached the same conclusion that northern populations of *M. integrifolia* were the wild origin of macadamia domestication^15^.

The extraordinary hard-shell of the macadamia fruit is a distinctive feature of the genus and is likely to have impeded international domestication of the crop until more efficient mechanical cracking methods were developed^34^. MADS-box transcription factors *TT16* and *STK* are the major regulators of cell orientation and differentiation during the formation of the integument from which the seed coat develops^35,36^. PAs, a class of phenylpropanoid metabolites that play an important role in seed development, are regulated by *MiTT16*, *MiSTK*, *MiTT2,* and other transcription factors^24^. Lignin is one of the main components of the macadamia seed coat and contributes to hardness, stiffness, and strength in seed coat^19^. According to current evidence, peroxidases (CIIIPRXs; EC 1.11.1.7) and laccase (EC 1.10.3.2) are responsible for monolignol oxidation and coupling ultimately the formation of lignin^23,37,38^. In the macadamia genome, an expansion of *MiSTK*, *MiTT16,* and *MiPRX17* gene families (Fig. 3e, f), suggests these genes may play a major role in seed coat development. The expression of *STK*, *TT16*, *PRX17,* and *AGL15* across all tested tissues further indicates their essential functions in macadamia plant development (Supplementary Figs. 8, 9 and Supplementary Table 17). Expansion of these genes likely contributed to the evolution of the hard shell in macadamia (Fig. 3d).

Ketoacyl synthases (KAS) and stearoyl-ACP desaturase (SAD/FAB2) are two key enzymes for fatty acid elongation of enoyl-ACP (4:0-ACP) to palmitoyl-ACP (16:0-ACP) and desaturation of stearic acid (18:0) to oleic acid (18:1)^39^. Compared with other species, *MiKASI* and *MiFAB2* are expanded in the macadamia genome (Fig. 3a, b). These expanded copies may have different functions between them and exhibit substrate specificities in different tissues^40,41^. The expression of *KASI* and *FAB2* in different development stages of macadamia kernel development support that these two genes played a major role in the fatty acid biosynthesis during kernel development (Fig. 4d). Notably, genes related to the fatty acid biosynthesis (TFs^42^ *MiABI3*, *MiWRI,* and *MiFUS*) were highly expressed in the kernel (Fig. 4e). This indicates that fatty acid biosynthetic pathways are critical for domestication and maintaining high oil content in kernels.

Many wild plant species in the wild have the potential to be domesticated for food, fuel, fiber, and medicine. Macadamia has a long generation time and has been subject to a few generations of selective breeding over <100 years of domestication. Despite this, the success of macadamia commercialization demonstrates that rapid domestication of new crops is feasible. The detection of selective sweeps in Hawaiian macadamia cultivars that are two generations removed from wild ancestors provides insights into the genomic basis for such accelerated domestication. The continuous advancement of genomic technologies is increasingly making sequencing and re-sequencing plant genomes a routine practice. Genotyping by genome re-sequencing coupled with phenotyping of a large collection of elite germplasm after initial evaluation, it is achievable to shorten the time required for domestication and utilization of wild germplasm and plant resources.

## Methods

### Sample collection and DNA sequencing

‘Kau’ (HAES 344), a popular cultivar developed in Hawaii from the species *M. integrifolia*, was used for de novo genome assembly. An additional 112 accessions presumed to be from the same species were used for whole genome re-sequencing and population genetics analysis (Supplementary Table 21). Leaf samples for 55 accessions (including HAES 344) were collected and provided by the United States Department of Agriculture Agricultural Research Service from reference plants for each accession in the Hilo, Hawaii germplasm repository. South Subtropical Crop Research Institute, China Academy of Tropical Agricultural Sciences provided material for an additional 15 accessions. Southern Cross University, Australia provided DNA samples isolated from 42 wild accessions of *M. integrifolia*, which were collected from Alstonville, Tiaro, and Burpengary in eastern Australia. These collected from wild populations with permission from private landholders or from the National Macadamia Germplasm Collection.

PacBio RSII sequencing combined with Hi-C (High-throughput chromosome conformation capture) assisted genome assembly technologies were used for chromosomal-level assembly of the HAES 344 reference genome. For PacBio long-reads sequencing, BluePippin systems were used for size selection. 20-kb SMRTbell libraries were prepared according to the released protocol from PacBio and 70 Single-Molecule, Real-Time (SMRT) cells were sequenced on a PacBio RS II system. For Hi-C libraries construction^43^, about 1–2 g young leaves were prepared for cells fix by formaldehyde with 1% formaldehyde solution in MS buffer, following nuclei extraction, nuclei permeabilization, chromatin digestion (DpnII), and proximity ligation treatments, the final constructed libraries were sequenced on the NovaSeq platform. The DNA extracts used for whole genome re-sequencing were sequenced using Illumina NovaSeq platform at ~20× genomic coverage with 150-bp read length and 300–500 bp insert size.

### Flow cytometry

Fresh young leaves were used for sample preparation. Cell nuclei were released from a small amount of fresh plant tissue by mechanical homogenization in MGb buffer, following filtration by 400T mesh. 10,000 nuclei were isolated based on fluorescently labeled propidium iodide in a single experiment, and the cross-validation (CV)% was controlled below 5%. Nuclei were surveyed by BD FACScalibur with gating through SSC vs. FL.

For each measurement, the propidium iodide fluorescence area signals (FL2-A) from 1000 nuclei were collected and analyzed by CellQuest software (Becton-Dickinson, San Jose, CA). The mean position of the G0/G1 (Nuclei) peak of the sample and the internal standard were determined by CellQuest software. The mean nuclear DNA content of each plant sample, measured in picograms, was based on 1000 scanned nuclei. One biological sample was taken from each accession, and for each biological sample, four technical replications were done, and the average was used as the representative genome size. Details of the method and calculations are described in Dolezel et al. (2007)^44^.

### Genome assembly

PacBio single-molecule real-time (SMRT) long-reads sequences, Illumina short-read sequences as well as high throughput chromatin conformation capture (Hi-C) technologies were combined to assembly the macadamia genome. A total of 110 Gb (~110 × coverage, based on flow cytometry genome size estimate) of PacBio long read data were de novo assembled using CANU^45^ (version 1.9). An additional 50 Gb (~50× coverage) of Illumina pair-end short reads were used to further correct systematic errors of PacBio sequencing using Pilon software^46^. After CANU assembly, Benchmarking Universal Single-copy Orthologs (BUSCO) (v3.0.2, embryophyta_odb9) was used to evaluate genome completeness and duplication score^47^. PurgeHaplotigs^48^ were used to remove the genome duplication. Misassembled contigs of the initial CANU assembly were identified and corrected based on Hi-C sequencing using juicer tools^49^ and the 3D-DNA pipeline^50^. The corrected contigs were then partitioned into 14 groups, representing 14 pseudo-chromosomes by ALLHiC software^51^.

### Genome annotation

Genome annotation and gene prediction were undertaken using two rounds of MAKER after training the program using *M. integrifolia* expressed genes^52^. Prior to gene prediction and annotation, repeatMasker (http://www.repeatmasker.org/) and Teclassify^53^ were used to annotate repetitive sequences. Kimura distances were calculated by sub-program (createRepeatLandscape.pl) of repeatmasker. Transcripts and ORFs were constructed using combination of HISAT2^54^, Stringtie^55^, and PASA^56^. The ab initio prediction of protein-coding genes was carried out by the MAKER^57^. Functional annotation of protein-coding genes was evaluated based on three protein sequence databases—SwissProt, InterPro^58^ and Pfam, and gene ontology (GO) information was obtained from the corresponding InterPro or Pfam entry. BUSCO was used for the evaluation of annotation completeness.

### Synteny analyses

*Macadamia* and sacred lotus *Nelumbo* belong to the same basal eudicot order Proteales. For comparative genome analyses of *M. integrifolia* and *N. nucifera*, MCscan^59^ was used to identify and plot the syntenic blocks. Wgd simple command line tools for the analysis of ancient WGDs^60^ was used to distinguish paralogues and orthologues genes between and in *M. integrifolia* and *N. nucifera* genome, and to estimate synonymous divergence levels (*K*_s_). WGD time was estimated by combining the *K*_s_ value with synonymous substitutions at each site per year (*r*) through Eq. (1)

1 $${{{{{\rm{divergence}}}}}}\,{{{{{\rm{date}}}}}}\,({T})=K_{\rm {{s}}}/2r$$

### Phylogenetic reconstruction and gene family expansion/contraction

Single-copy orthologous genes were identified by using OrthoMCL^61^ for the *M. integrifolia* genome and six other plant species (*A. thaliana*, *O. sativa*, *S. lycopersicum*, *N. nucifera*, *V. vinifera*, *C. papaya*), which were downloaded from Phytozome (https://phytozome.jgi.doe.gov/pz/portal.html). A maximum-likelihood phylogenetic tree, based on multiple sequence alignment of amino acid sequences of single-copy genes from these seven species, was constructed by RaxML^62^. The MCMCTree program in the PAML package^63^ was used to estimate the species divergence times using the *A. thaliana* and *C. papaya* divergence time (68–72 million years ago)^12^ and the monocot and eudicot divergence time (120–140 million years ago)^12^ as calibrators. CAFE^64^ was used to calculate the expansion and contraction of gene family numbers based on the phylogenetic tree and gene family statistics family-wise error rate based on a Monte-Carlo re-sampling procedure.

### RNA sequencing and analysis

Tissues of leaf, stem, flower, and root for RNA sequencing were collected from cultivar ‘Kau’ (HAES 344), and five development stages of shell and kernel were collected from the cultivar ‘Hinde’ (‘H2’). Each development stage of shell and kernel was sampled in three replicates. Total RNA was extracted using the TaKaRa MiniBEST Plant RNA Extracting Kit according to the manufacturer’s instructions. RNA quality and quantity were further assessed using NanoDrop 2000C and Agilent 2100 platforms. Sequencing libraries were generated using NEBNext^®^ Ultra™ RNA Library Prep Kit following the manufacturer’s recommendations and then sequenced on the Illumina Hiseq2500 platform to generate 150 bp paired-end reads, and finally yielding 5 Gb data for each sample. Before alignment, reads were trimmed to remove the adaptors and low-quality bases by using trimmomatic program^65^. FPKM (fragments per kilobase of exon per million fragments mapped) were generated by using the RSEM^66^ package of Trinity^67^. Further, the DEGs were identified using DESeq2^68^.These DEGs were also analyzed by WGCNA^69^, an R package for gene co-expression network analysis. The R packages mfuzz^70^ was used to make DEG clusters. GO enrichment and KEGG pathway analysis was performed using the OmicShare tools (http://www.omicshare.com/tools), a free online platform for data analysis.

### Population genomic analysis

All re-sequencing reads from macadamia cultivars, landraces, and wild relatives (~20 × genomic coverage) were mapped to the *M. integrifolia* ‘Kau’ (HAES 344) reference genome assembly using the Mem module in BWA with default parameters^71^. SNPs and small indels (1–10 bp) were identified using the Genome Analysis Toolkit (GATK) pipeline^72^. To ensure variant accuracy, unique mapping, and IndelRealigner were applied to process the alignment BAM files. VCF files for each sample were produced by HaplotypeCaller and finally merged to a single VCF file by GATK Genotype GVCFs function. SNPs were filtered to remove low-quality variants with vcftools^73^ using the following rules: Two alleles with coverage depth of 4–60×, minor allele quality of 30, minor allele frequency of 5%, maximum missing data of 10%.

Population structure was initially examined by constructing a phylogenetic tree using the maximum-likelihood (ML) method implemented in iqtree^74^ and displayed in iTOL^75^. The optimum number of sub-populations (*K*) was estimated by identifying the *K* value at which cross-validation was minimized across the range of 1–10 using the program Admixture^76^. GCTA^77^ were used for PCA analysis with input Plink binary files, which were transformed from the filtered VCFs file using VCFtools (v0.1.13) and PLINK (v1.07)^78^. The top two principal components were used for assigning the accessions and downstream population structure analysis. Pairwise population differentiation (*F*_ST_) and nucleotide diversity (*π*) were estimated by PLINK^78^ and VCFtools using genome-wide high-quality SNPs with 50-kb sliding window and 20-kb steps. TreeMix^79^ was used to determine the historical relationships between Hawaiian cultivars and three wild groups, and gene flow between them.

Signals of selective sweeps in the macadamia genome during its short history of domestication were identified by population fixation statistics (*F*_ST_) and implemented in the program XP-CLR^80^ using the likelihood method for detecting selective sweeps between two populations. A total of 9,541,414 high-quality SNPs were subjected to XP-CLR xpclr with 50-kb sliding window and 20-kb step for each chromosome. The top 5% XP-CLR values across the genome were considered be potential selected loci. Candidate selective sweeps were further narrowed using *F*_ST_, which was calculated in a 50-kb sliding window and a 20-kb step using VCFtools. The overlap 387 blocks were also narrowed by Tajima’s *D* (negative in varieties and positive in wild). Candidate regions in the top 5% of distribution for *F*_ST_ values overlapping with XP-CLR regions and Tajima’s *D* were considered as the final set of selective sweeps in the *M. integrifolia* genome.

Site frequency spectrum (SFS) for each sample were estimated using ANGSD^81^. The bam files generated from read mapping of accessions were filtered when running the ANGSD (only use reads where the mate could be mapped, discards reads that do not map uniquely, discard bases with base quality below 20 and with mapping quality below 30). The SFS was then used for estimating the population demography history using software Stairway plots^82^ with 200 bootstrap iterations. The mean generation time was set at 8 years, the age at which >90% of trees produce fruit^83^. As there are no previous estimates of mutation rate for macadamia, mutation rate of *N. nucifera* (mu = 4.175e–9) from the same order Proteales was used when estimating the demography history by Stairway plots analysis^84^.

### Reporting summary

Further information on research design is available in the Nature Research Reporting Summary linked to this article.

## Supplementary information

Supplementary Information

Reporting Summary

## Data Availability

The Macadamia integrifolia genome project data has been deposited at the NCBI under the BioProject ID PRJNA706119. The PacBio RS II sequencing data were deposited in the Sequence Read Archive database under the accession number SAMN18118504. The Macadamia integrifolia genome assemblies, gene sequences, and annotation data are also available at China National Center for Bioinformation [https://ngdc.cncb.ac.cn/gwh/Assembly/23196/show]. Source data are provided with this paper.

## Source data

Source Data

## Code availability

Softwares and parameters use in this paper are listed in GitHub [https://github.com/lvvn/genome-analysis].
