# Supplementary material for: Signatures of selection in recently domesticated macadamia
Source: Nat Commun. 2022 Jan 11;13:242. doi: 10.1038/s41467-021-27937-7 (PMC8752631; doi:10.1038/s41467-021-27937-7)
Supplement: Supplementary file 1 — Supplementary Information [file 41467_2021_27937_MOESM1_ESM.pdf]

Supplementary Information

Signatures of selection in recently domesticated macadamia

Lin *et al*.

Supplementary Table 1. Flow cytometry result of genome size

Fluorescence intensity		Ratio	Size(G）	Average	SD
O.sativa	*M.integrifolia*				
21.73	45.68	2.10	0.88		
21.49	45.48	2.12	0.89	0.89	0.01
21.65	46.11	2.13	0.89		

Supplementary Table 2. Sequencing information

Items	Sequencing platform	
	**PacBio RSII**	**Illumina NovaSeq**
Total Number of reads	8568299	166031735
Total Number of sequenced Bases (Gb)	89.2	46.4
Mean reads length (bp)	11174	150
N50 (bp)	14374	150
Coverage (X)	~99	~51

Supplementary Table 3. Statistics of contig-level genome assembly and annotation

Items	Contig level assembly(CANU)	After eliminating redundant sequences
**Assembly size (bp)**	**1098262641**	**793595930**
**No. of contigs**	**15450**	**5387**
**Maximum length (bp)**	**3233473**	**3233473**
**N90 (bp)**	**26667**	**61527**
**N80 (bp)**	**40498**	**107049**
**N70 (bp)**	**62162**	**159225**
**N60 (bp)**	**101932**	**214567**
**N50 (bp)**	**163467**	**280713**
**Average length (bp)**	**71084**	**147316**

Supplementary Table 4. BUSCO completeness analysis of genome assembly and annotation

Descripation	Genome		Annotation	
	Number	Percentage(%)	Number	Percentage(%)
**Complete BUSCOs (C)**	1266	92.1	1216	88.4
**Complete and single-copy BUSCOs (S)**	1122	81.6	1074	78.1
**Complete and duplicated BUSCOs (D)**	144	10.5	142	10.3
**Fragmented BUSCOs (F)**	43	3.1	105	7.6
**Missing BUSCOs (M)**	66	4.8	54	4.0
**Total BUSCO groups searched**	1375	100.0	1375	100.0

Supplementary Table 5. chromosome scale of genome assembly

ChrID	No. Of anchored contigs	Length(bp)
**Chr1**	548	63679829
**Chr2**	597	61630830
**Chr3**	278	72930807
**Chr4**	459	57852922
**Chr5**	403	54274071
**Chr6**	368	53551794
**Chr7**	314	56508535
**Chr8**	373	51452572
**Chr9**	357	63039168
**Chr10**	382	49445334
**Chr11**	375	49140984
**Chr12**	372	47947681
**Chr13**	240	60251108
**Chr14**	309	52188577
**Total number of contigs**		5387
**Total length of contigs**		793595930
**Total number of anchored contgis**		5375
**Total length of chromosome level assembly**		793894212
**Number of unanchored contigs**		12
**Length of unanchored contigs**		237818
**Anchor rate (%)**		99.97
**No. of Protein-coding genes**		37728
**No. of known miRNAs**		113

Supplementary Table 6. Assessment of genome assemblies based on RNA-seq assembled transcripts

	Number	Total Length (bp)	Accuracy (%)	Bases covered by assembly (%)	Sequences covered by assembly (%)	With >90% sequence in same chromosome		With >50% sequence in same chromosome	
						Number	Percent(%)	Number	Percent(%)
RNA assembled transcripts	218893	572642300	99.99	99.99	99.99	218880	99.99	218880	99.99

Supplementary Table 7. Assessment of genome consistency based on illumina reads

Iterms	
**Number of reads**	166031735
**Data size(Gb)**	46.39
**Mapping rate(%)**	99.52
**Genome Length(Mbp)**	794
**Mean Depth**	58.71
**Coverage Rate(%)**	99.03

Supplementary Table 8. TE annotation

	Length(M)	% of genome
**Total repeat fraction**	461.07	56.97
**Class I: Retroelement**	396.36	48.98
**LTR Retrotransposon**	266.86	32.97
Ty1/Copia	51.87	6.41
Ty3/Gypsy	133.49	16.49
Other	81.50	10.07
**Non-LTR Retrotransposon**	100.86	12.46
LINE	92.89	11.48
SINE	7.97	0.98
**Unclassified retroelement**	28.65	3.54
**Class II: DNA Transposon**	71.46	8.83
**TIR**		
CMC	1.37	0.17
hAT	17.69	2.19
Mutator	4.99	0.62
Tc1/Mariner	0.23	0.03
PIF/Harbinger	3.37	0.42
Other	43.57	5.38
Helitron	1.18	0.15
**Tandem Repeats**	31.65	3.91
**Unkown**	8.52	1.05

Supplementary Table 9. Overall of orthogroups in 6 species

Number of species	6
Number of genes	213308
Number of genes in orthogroups	143841
Number of unassigned genes	69467
Percentage of genes in orthogroups	67.4
Percentage of unassigned genes	32.6
Number of orthogroups	14999
Number of species-specific orthogroups	396
Number of genes in species-specific orthogroups	2485
Percentage of genes in species-specific orthogroups	1.2
Mean orthogroup size	9.6
Median orthogroup size	8
G50 (assigned genes)	11
G50 (all genes)	8
O50 (assigned genes)	4110
O50 (all genes)	7911
Number of orthogroups with all species present	8955
Number of single-copy orthogroups	898

Supplementary Table 10 Statistics of orthogroups in PerSpecies

	Arabidopsis	Grape	Macadamia	Rice	Sacred_Lotus	Tomato
Number of genes	35386	26346	37742	52424	26685	34725
Number of genes in orthogroups	26719	19222	26889	26239	20927	23845
Number of unassigned genes	8667	7124	10853	26185	5758	10880
Percentage of genes in orthogroups	75.5	73	71.2	50.1	78.4	68.7
Percentage of unassigned genes	24.5	27	28.8	49.9	21.6	31.3
Number of orthogroups containing species	12164	12435	13183	11898	12513	12876
Percentage of orthogroups containing species	81.1	82.9	87.9	79.3	83.4	85.8
Number of species-specific orthogroups	81	26	42	186	16	45
Number of genes in species-specific orthogroups	550	143	244	1180	67	301
Percentage of genes in species-specific orthogroups	1.6	0.5	0.6	2.3	0.3	0.9

Supplementary Table 11 Gene numbers in every cluster of Mfuzz ananlysis

ClusterID	Counts
cluster1	96
cluster2	453
cluster3	110
cluster4	164
cluster5	404
cluster6	140
cluster7	231
cluster8	81
cluster9	202
cluster10	374
cluster11	130
cluster12	473
cluster13	283
cluster14	41
cluster15	312
cluster16	350

Supplementary Table 12. RPKM values of cluster16 genes

	Shell1	Shell2	Shell3	Shell4	Shell5	Kernel1	Kernel2	Kernel3	Kernel4	Kernel5	Leaf	Flower	Stem	Root
Mi03Gene55932	15	0	38	19	5	0	0	0	5	10	7	0	6	0
Mi03Gene55933	2	2	7	3	5	0	0	0	0	1	1	0	1	2
Mi03Gene16345	7	6	8	9	8	1	0	0	1	1	2	1	7	3
Mi03Gene49456	7	7	10	11	12	0	0	0	1	0	1	0	5	3
Mi03Gene56826	2	6	7	9	10	0	0	3	1	1	0	1	1	1
Mi03Gene15354	2	4	6	4	8	0	0	0	0	0	1	2	1	2
Mi03Gene71841	6	7	11	9	14	0	0	0	0	0	2	1	5	4
Mi03Gene42314	17	17	16	19	20	5	8	8	14	12	5	2	21	11
Mi03Gene27411	12	12	15	15	14	1	7	6	5	5	3	1	12	9
Mi03Gene61731	96	49	68	61	118	0	0	31	19	16	14	15	22	39
Mi03Gene37911	16	27	38	38	44	1	1	5	9	6	3	2	10	9
Mi03Gene68915	8	4	5	0	20	0	0	0	0	0	1	0	3	0
Mi03Gene15921	0	6	7	5	4	0	0	0	0	0	0	0	0	0
Mi03Gene15925	0	3	12	6	11	0	0	0	0	0	0	2	9	0
Mi03Gene06140	0	1	3	5	7	1	0	0	1	0	0	0	0	0
Mi03Gene06143	0	0	1	2	2	0	0	0	1	0	0	0	0	0
Mi03Gene57789	257	313	430	401	477	9	3	4	5	5	77	634	109	12
Mi03Gene51983	10	12	24	18	16	0	0	0	0	0	6	4	8	20
Mi03Gene27708	290	386	412	484	495	29	70	115	16	130	46	23	65	86
Mi03Gene17136	70	143	123	137	189	105	26	15	19	11	6	59	112	26
Mi03Gene15665	1,183	1,690	2,188	2,540	2,628	216	191	182	407	477	284	293	597	390
Mi03Gene67333	146	174	195	208	229	55	31	53	32	19	14	21	23	60
Mi03Gene67343	147	342	373	365	405	223	126	86	81	36	3	152	30	5
Mi03Gene04926	28	43	73	59	68	5	0	0	3	2	0	0	0	20
Mi03Gene70347	5	7	11	11	11	2	1	3	6	6	2	1	3	4
Mi03Gene06636	19	32	52	50	72	2	3	11	7	4	30	2	22	8
Mi03Gene49469	549	791	899	937	1,032	11	5	4	3	23	0	9	2	38
Mi03Gene63800	2	3	12	4	8	0	0	0	1	1	0	0	7	3
Mi03Gene69745	46	66	98	115	130	4	5	6	9	15	6	12	110	46
Mi03Gene27647	2,755	4,277	4,998	5,944	8,316	253	796	1,156	1,199	1,493	414	286	837	986
Mi03Gene71293	35	58	62	78	71	4	21	24	6	6	54	39	15	10

Supplementary Table 13. Functions of cluster16 genes

Gene IDs	Orthologous	Short names	Function
Mi03Gene55932	AT2G18060	VND1, NAC1	secondary cell wall biosynthesis
Mi03Gene55933	AT2G18060	VND1, NAC1	secondary cell wall biosynthesis
Mi03Gene16345	AT2G18060	VND1, NAC1	secondary cell wall biosynthesis
Mi03Gene49456	AT1G12260	NAC7,VND4	secondary cell wall formatioin.
Mi03Gene56826	AT1G66370	MYB113	anthocyanin biosynthesis
Mi03Gene15354	AT1G66370	MYB113	anthocyanin biosynthesis
Mi03Gene71841	AT1G09540	MYB61	phloem or xylem histogenesis
Mi03Gene42314	AT1G52150	ATHB-15	vascular development
Mi03Gene27411	AT5G61480	PXY	phloem or xylem histogenesis
Mi03Gene61731	AT3G28455	CLE25	cell fate commitment
Mi03Gene37911	AT5G19530	ACL5	vascular development
Mi03Gene68915	AT4G36890	IRX14	phloem or xylem histogenesis,<br>xylan biosynthesis
Mi03Gene15921	AT4G21760	BGLU47	coniferin metabolism
Mi03Gene15925	AT4G21760	BGLU47	coniferin metabolism
Mi03Gene06140	AT5G09730	BXL3	xylan 1,4-beta-xylosidase activity
Mi03Gene06143	AT5G09730	BXL3	xylan 1,5-beta-xylosidase activity
Mi03Gene57789	AT1G67980	CCOAMT	lignin biosynthsis
Mi03Gene51983	AT5G03260	LAC11	lignin biosynthsis
Mi03Gene27708	AT5G59030	COPT1	pollen sperm cell differentiation
Mi03Gene17136	AT4G38400	EXLA2	plant-type cell wall organization
Mi03Gene15665	AT2G39700	EXPA4	plant-type cell wall organization
Mi03Gene67333	AT1G53840	PME1	pectin cellwall formation
Mi03Gene67343	AT4G33220	PME44	pectin cellwall formation
Mi03Gene04926	AT5G53190	SWEET3	sugar transporter
Mi03Gene70347	AT3G63430	TRM5	
Mi03Gene06636	AT1G11545	XTH8	
Mi03Gene49469	AT1G62770	PMEI9	pectin methyleseterase inhibitor
Mi03Gene63800	AT5G66390	peroxidase	
Mi03Gene69745	AT4G13710		pectin catabolic process
Mi03Gene27647	AT5G62350		pectin methyleseterase inhibitor;<br>cellwall formation
Mi03Gene71293	AT4G02320		cell wall modification

Supplementary Table 14. RPKM values of cluster5 genes

	Shell1	Shell2	Shell3	Shell4	Shell5	Kernel1	Kernel2	Kernel3	Kernel4	Kernel5	Leaf	Flower	Stem	Root
Mi03Gene44588	5	3	4	3	4	1	1	0	0	1	0	0	0	0
Mi03Gene44591	3	3	3	2	2	0	0	0	0	0	0	0	0	0
Mi03Gene20859	49	50	49	40	39	2	10	9	9	8	18	0	5	1
Mi03Gene05164	676	869	606	711	655	25	1	3	10	7	80	14	150	364
Mi03Gene28092	10	15	10	11	13	3	3	1	4	2	7	4	5	1
Mi03Gene65648	112	131	98	122	117	2	1	4	10	14	21	0	9	0
Mi03Gene64510	724	756	587	631	672	13	0	0	8	14	0	50	0	0
Mi03Gene64499	66	33	49	54	24	2	0	0	0	2	0	0	0	0
Mi03Gene22856	66	66	54	73	52	2	0	0	1	0	15	2	9	15
Mi03Gene22852	66	66	54	73	52	2	0	0	1	0	15	2	9	15
Mi03Gene15795	628	634	455	436	421	112	0	4	50	4	0	2	1	0
Mi03Gene58576	167	196	139	155	155	35	1	2	11	2	0	1	0	0
Mi03Gene08879	242	217	186	190	193	8	1	1	4	1	2	151	3	0
Mi03Gene27849	329	255	191	218	251	15	1	1	9	5	0	113	0	0
Mi03Gene71176	5	11	12	8	4	1	0	0	1	0	0	0	0	0
Mi03Gene71177	11	21	20	19	17	4	1	1	3	2	0	0	2	1
Mi03Gene61413	195	247	139	250	213	20	1	0	15	1	0	39	0	0
Mi03Gene33834	374	482	435	585	440	16	5	3	5	4	91	98	81	180
Mi03Gene66470	701	641	605	661	664	176	226	217	241	217	153	227	598	279
Mi03Gene54732	368	450	375	393	357	154	61	28	11	14	41	22	54	170
Mi03Gene17506	2,306	2,852	2,568	2,941	2,643	88	15	11	31	37	477	170	245	929
Mi03Gene54727	928	1,222	1,173	1,479	1,264	450	118	89	37	42	102	55	142	492
Mi03Gene65002	161	212	168	205	171	60	13	7	2	2	27	45	26	116
Mi03Gene48079	162	193	151	196	139	7	0	1	3	2	22	13	19	55
Mi03Gene70646	64	56	49	57	58	6	19	21	29	25	8	21	18	21
Mi03Gene71237	923	994	592	649	449	23	10	4	5	3	146	129	174	431
Mi03Gene12065	1,275	1,415	1,054	1,301	966	41	2	5	12	10	260	52	147	508
Mi03Gene36831	185	171	174	152	148	8	15	95	80	69	21	14	13	2
Mi03Gene36751	3,424	3,631	2,754	3,185	2,398	97	10	12	28	16	369	473	263	815
Mi03Gene45377	45	74	41	58	49	12	2	2	11	1	3	4	32	41
Mi03Gene12338	118	89	118	133	117	2	0	1	2	2	0	9	0	0
Mi03Gene12346	111	86	69	72	72	1	0	0	1	1	0	11	0	0
Mi03Gene12341	372	327	213	205	188	13	2	2	9	5	40	15	29	2
Mi03Gene12343	8,967	7,977	7,425	9,451	8,908	258	24	55	162	113	1,598	282	1,105	28
Mi03Gene67345	39	35	23	39	33	4	7	11	22	29	8	1	13	24
Mi03Gene06655	1,138	1,437	1,594	1,761	1,836	30	10	13	22	19	135	31	145	178
Mi03Gene14543	7	14	6	14	6	0	0	0	0	0	0	1	7	1
Mi03Gene07029	1,142	1,136	911	1,328	1,788	78	89	149	81	66	257	51	211	49

Supplementary Table 15. Functions of cluster5 genes

Gene IDs	Orthologous	Short names	Function
Mi03Gene44588	AT1G69560	MYB105	
Mi03Gene44591	AT1G69560	MYB105	
Mi03Gene20859	AT5G49330	MYB111	
Mi03Gene05164	AT5G49330	MYB111	
Mi03Gene28092	AT3G61250	MYB17	
Mi03Gene65648	AT5G52600	MYB82	
Mi03Gene64510	AT4G09960	STK	
Mi03Gene64499	AT4G09960	STK	
Mi03Gene22856	AT3G59030	TT12	
Mi03Gene22852	AT3G59030	TT12	
Mi03Gene15795	AT5G23260	TT16	
Mi03Gene58576	AT5G23260	TT16	
Mi03Gene08879	AT4G18960	AG	
Mi03Gene27849	AT2G45650	AGL6	
Mi03Gene71176	AT2G35740	INT3	sugar transporter
Mi03Gene71177	AT4G16480	INT4	sugar transporter
Mi03Gene61413	AT5G23660	SWEET12	sugar transporter
Mi03Gene33834	AT2G37040	PAL1	phenylpropanoid
Mi03Gene66470	AT4G29080	PAP2	phenylpropanoid
Mi03Gene54732	AT3G51240	F3H	phenylpropanoid
Mi03Gene17506	AT3G51240	F3H	phenylpropanoid
Mi03Gene54727	AT3G51240	F3H	phenylpropanoid
Mi03Gene65002	AT3G21240	4CL2	phenylpropanoid
Mi03Gene48079	AT1G65060	4CL3	phenylpropanoid
Mi03Gene70646	AT5G19530	ACL5	phenylpropanoid
Mi03Gene71237	AT5G13930	TT4，CHS	phenylpropanoid
Mi03Gene12065	AT5G07990	TT7, F3'H	phenylpropanoid
Mi03Gene36831	AT2G30370	CHAL	flavonoid biosynthesis
Mi03Gene36751	AT5G05270	CHIL	flavonoid biosynthesis
Mi03Gene45377	AT5G50260	CEP1	secondary wall thickening
Mi03Gene12338	AT2G44490	PEN2	phenylpropanoid
Mi03Gene12346	AT2G44490	PEN2	phenylpropanoid
Mi03Gene12341	AT2G44490	PEN2	phenylpropanoid
Mi03Gene12343	AT2G44490	PEN2	phenylpropanoid
Mi03Gene67345	AT1G53830	PME2	pectin methyleseterase inhibitor
Mi03Gene06655	AT1G05260	RCI3	phenylpropanoid biosynthesis
Mi03Gene14543	AT2G36870	XTH32	xyloglucan metabolic process
Mi03Gene07029	AT4G03210	XTH9	xyloglucan metabolic process

Supplementary Table 16. Selected cis-elements present in the 1500-bp sequence upstream of the Prx17 gene in macadamia

Gene ID	Site name	Cis-element	Function	Position	Similar Score
MiPRX17A	CARGCW8	CAATTtaaag	Putative binding Site for MaAGL15	-215	1
MiPRX17B	MYBPLANT	cACCAAcc	Related to lignin biosynthesis	-16	1

Supplementary Table 17. Chromosomal locations of *STK*, *PRX17* and *KASI* paralogous genes.

ID	Gene Name	Chromosome	Start	End
mi03Gene52846	*MiSTK2*	Chr6	15174848	15191840
mi03Gene64499	*MiSTK1*	Chr8	17403066	17429308
mi03Gene31522	*MiKASI.1*	Chr2	11313625	11325793
mi03Gene31515	*MiKASI.2*	Chr2	11286792	11297539
mi03Gene19364	*MiKASI.3*	Chr12	30837834	30859978
mi03Gene57579	*MiKASI.4*	Chr7	7900886	7905127
mi03Gene57580	*MiKASI.5*	Chr7	7908949	7913039
mi03Gene12162	*MiKASI.6*	Chr11	9671486	9674796
mi03Gene51593	*MiPrx17A*	Chr6	5088315	5089483
mi03Gene63504	*MiPrx17B*	Chr8	9545525	9546577

Supplementary Table 18. Statistics of expanded and contracted genes in six species

Species	Expanded	fams	Genes/ gained	genes/ expansion	Contracted	fams	Genes/ lost	Genes/ contraction	No change	Avg.Expansion
At	2144	89	4116	1.92	2866	19	3392	1.18	9289	0.0506329
Os	1997	241	5259	2.63	4045	41	4787	1.18	8257	0.0330093
Lotus	1880	75	2922	1.55	1860	15	2175	1.17	10559	0.0522414
Tomato	2363	153	5253	2.22	2017	7	2311	1.15	9919	0.205749
Vv	1199	103	2541	2.12	4136	21	4937	1.19	8964	-0.167564
Maca	3955	269	6699	1.69	1704	16	2007	1.18	8640	0.328135

Supplementary Table 19. Pathway enrichment of expanded genes in *M. integrifolia* genome

The statistical tests were one-sided and Benjamini – Hochberg was used when adjustments were made for multiple comparisons.

Term	Database	ID	Input	Background number	Pvalue	Corrected Pvalue
benzoate-biosynthesis-I	BioCyc	PWY-6443	21	39	3.14E-11	8.45E-10
glucosinolate-biosynthesis	BioCyc	PWY-2821	12	12	4.29E-09	9.26E-08
chitin-degradation-II	BioCyc	PWY-6902	11	11	1.93E-08	3.76E-07
acetaldehyde-biosynthesis-I	BioCyc	PWY-6333	10	13	4.53E-07	6.83E-06
pyruvate-fermentation-to-ethanol-II	BioCyc	PWY-5486	10	13	4.53E-07	6.83E-06
ethanol-degradation-II	BioCyc	PWY66-21	10	19	5.62E-06	6.30E-05
cis-vaccenate-biosynthesis	BioCyc	PWY-5973	8	9	3.20E-06	3.85E-05
fatty-acid-elongation-saturated	BioCyc	FASYN-ELONG-PWY	8	10	5.45E-06	6.13E-05
stearate-biosynthesis-II	BioCyc	PWY-5989	8	11	8.90E-06	9.61E-05
palmitate-biosynthesis-II	BioCyc	PWY-5971	8	12	1.40E-05	1.45E-04
superpathway-of-fatty-acid-biosynthesis-II	BioCyc	PWY-5156	8	21	2.89E-04	2.06E-03
callose-biosynthesis	BioCyc	PWY-6773	7	13	1.33E-04	1.06E-03
photorespiration	BioCyc	PWY-181	7	14	1.89E-04	1.42E-03
eugenol-and-isoeugenol-biosynthesis	BioCyc	PWY-5859	5	6	3.1E-04	2.19E-03
indole-3-acetate-biosynthesis-II	BioCyc	PWY-581	7	18	6.29E-04	4.13E-03
pyridine-nucleotide-cycling	BioCyc	PWY-5381	7	19	8.14E-04	5.18E-03
Plant-pathogen-interaction	KEGG	ath04626	35	170	4.95E-08	9.22E-07
Glucosinolate-biosynthesis	KEGG	ath00966	13	23	1.18E-07	2.00E-06
Cyanoamino-acid-metabolism	KEGG	ath00460	20	69	4.20E-07	6.39E-06
Biosynthesis-of-secondary-metabolites	KEGG	ath01110	113	1107	1.46E-05	1.49E-04
Tyrosine-metabolism	KEGG	ath00350	12	40	6.43E-05	5.56E-04
Fatty-acid-degradation	KEGG	ath00071	12	47	2.32E-04	1.73E-03
Diterpenoid-biosynthesis	KEGG	ath00904	8	22	3.73E-04	2.54E-03
Cutin,-suberine-and-wax-biosynthesis	KEGG	ath00073	9	31	6.38E-04	4.17E-03
Basal-transcription-factors	KEGG	ath03022	12	55	7.87E-04	5.05E-03

Supplementary Table 20. Fatty acid biosynthesis related gene family numbers in 14 species

	GPAT9	DGAP	FAD		KAS		
			FAB/SAD	FAD	KASI	KASII	KASIII
Amborella trichopoda	1	2	4	5	1	1	0
Arabidopsis thaliana	1	2	8	15	1	1	1
Brassica rapa	2	4	7	32	1	4	1
Glycine max	4	8	4	18	4	3	2
Macadamia	2	5	12	11	6	2	2
Nelumbo nucifera	2	3	5	10	2	2	2
Oryza sativa	1	4	8	9	2	2	1
Slycopersicum	1	3	7	14	3	2	2
Vvinifera	1	3	7	8	2	2	2
Zea mays	2	4	4	17	2	3	1
Prunus dulcis	1	3	4	10	1	2	1
Corylus avellana	1	1	1	3	1	0	0
Arahis duranensis	2	6	6	11	1	2	3
Prunus dulcis	2	4	6	10	3	3	2

Supplementary Table 21. List of 112 macadamia cultivars and wild accessions used in genome re-sequencing and population genomic analyses

Sample NO.	Cultivar/line	HAES No.	Species	Source	Site Name of Sampling	Latitude	Longitude
M1	KAU	HAES344	*M.integrifolia*	Hawaii			
M2	KEAAU	HAES660	*M.integrifolia*	Hawaii			
M3	KEAUHOU	HAES246	*M.integrifolia*	Hawaii			
M4	MAKAI	HAES800	*M.integrifolia*	Hawaii			
M5	MAUKA	HAES741	*M.integrifolia*	Hawaii			
M6	PAHALA	HAES788	*M.integrifolia*	Hawaii			
M7	PURVIS	HAES294	*M.integrifolia*	Hawaii			
M9	KAKEA	HAES508	*M.integrifolia*	Hawaii			
M10	FAULKNER	HAES778	*M.integrifolia*	Hawaii			
M14	BURDICK	N95-57	*M.tetraphylla*	Hawaii			
M23	MCCORMACK	HAES920	*M.integrifolia*	Hawaii			
M24		N90-32	*M.integrifolia*	Hawaii			
M25	IAC-Camp-A	N92-03	*M.integrifolia*	Hawaii			
M26	IAC-2-23	N92-04	*M.integrifolia*	Hawaii			
M27	IAC-Camp-F	N92-06	*M.integrifolia*	Hawaii			
M29	IAC-CampH	N92-10	*M.integrifolia*	Hawaii			
M32	K-K-3	N95-42	*M.integrifolia*	Hawaii			
M34	D4	N95-24	*M.integrifolia*	Hawaii			
M38	JAMES	N96-18	*M.integrifolia*	Hawaii			
M39	SELECTX344	N96-19	*M.integrifolia*	Hawaii			
M40	SELECT#16	N96-20	*M.integrifolia*	Hawaii			
M41	N96-21	SELECT	*M.integrifolia*	Hawaii			
M43	N98-05	889	*M.integrifolia*	Hawaii			
M44	N12-10	857	*M.integrifolia*	Hawaii			
M45	N12-22	856	*M.integrifolia*	Hawaii			
M47	N13-17	Honokaa	*M.integrifolia*	Hawaii			
M48	M791	HAES791	*M.integrifolia*	Hawaii			
M49	M835	HAES835	*M.integrifolia*	Hawaii			
M50	s246		*M.integrifolia*	Hawaii			
M51	s335		*M.integrifolia*	Hawaii			
M52	s778		*M.integrifolia*	Hawaii			
M53	s783		*M.integrifolia*	Hawaii			
M54	s800		*M.integrifolia*	Hawaii			
M55	s831		*M.integrifolia*	Hawaii			
M56	s850		*M.integrifolia*	Hawaii			
M57	s857		*M.integrifolia*	Hawaii			
M58	s858		*M.integrifolia*	Hawaii			
M59	s863		*M.integrifolia*	Hawaii			
M60	s866		*M.integrifolia*	Hawaii			
M61	s879		*M.integrifolia*	Hawaii			
M62	s882		*M.integrifolia*	Hawaii			
M63	s887		*M.integrifolia*	Hawaii			

M64	s890		*M.integrifolia*	Hawaii			
M65	s894		*M.integrifolia*	Hawaii			
M66	s928		*M.integrifolia*	Hawaii			
M67	s930		*M.integrifolia*	Hawaii			
M68	sR13T5		*M.integrifolia*	Hawaii			
M71	HINDE		*M.integrifolia*	Australia			
M72	OWNCHOICE		*M.integrifolia*	Australia			
M73	NYS-1		*M.integrifolia*	China			
M74	NYS-2		*M.integrifolia*	China			
M75	NYS-3		*M.integrifolia*	China			
M76	NYS-4		*M.tetraphylla*	China			
M77	NYS-5		*M.tetraphylla*	China			
M78	NYS-6		*M.tetraphylla*	China			
M79	NYS-7		*M.integrifolia*	China			
M80	NYS-8		*M.integrifolia*	China			
M82	NYS-10		*M.integrifolia*	China			
M83	NYS-11		*M.integrifolia*	China			
M18	BEAUMONT	N95-56	M.hybrid	Hawaii			
M19	A16	N95-53	M.hybrid	Hawaii			
M21	HAES853	GREBERHYBRID	M.hybrid	Hawaii			
M22	PROBERT1	N95-54	M.hybrid	Hawaii			
M69	A4		M.hybrid	Australia			
M70	A16		M.hybrid	Australia			
M81	NYS-9		M.hybrid	China			
s1001-002	wild		*M.integrifolia*	Australia	MtBauple3	-25.821	152.585
s1001-003	wild		*M.integrifolia*	Australia	MtBauple3	-25.821	152.585
s1003-001	wild		*M.integrifolia*	Australia	MtBauple5	-25.807	152.585
s1003-004	wild		*M.integrifolia*	Australia	MtBauple5	-25.807	152.585
s1008-005	wild		*M.integrifolia*	Australia	AmamoorCk2	-26.359	152.646
s1009-003	wild		*M.integrifolia*	Australia	MaryRiver	-26.260	152.588
s1009-004	wild		*M.integrifolia*	Australia	MaryRiver	-26.260	152.588
s1009-006	wild		*M.integrifolia*	Australia	MaryRiver	-26.260	152.588
s1020-001	wild		*M.integrifolia*	Australia	MtNebo	-27.369	152.814
s1020-006	wild		*M.integrifolia*	Australia	MtNebo	-27.369	152.814
s1053-005	wild		*M.integrifolia*	Australia	MtBauple1	-24.390	151.370
s1055-002	wild		*M.integrifolia*	Australia	MaryCreek	-26.253	152.550
s1055-006	wild		*M.integrifolia*	Australia	MaryCreek	-26.253	152.550
s1058-003	wild		*M.integrifolia*	Australia	AmamoorCk1	-26.345	152.654
s1076-003	wild		*M.integrifolia*	Australia	Samford2	-27.342	152.818
s1076-005A	wild		*M.integrifolia*	Australia	Samford2	-27.342	152.818
s1076-005B	wild		*M.integrifolia*	Australia	Samford2	-27.342	152.818
s1077-006A	wild		*M.integrifolia*	Australia	Samford3	-27.393	152.820
s1077-006B	wild		*M.integrifolia*	Australia	Samford3	-27.393	152.820
W01-MB1	wild		*M.integrifolia*	Australia	MtBauple1	-24.390	151.370
W02-MB3	wild		*M.integrifolia*	Australia	MtBauple2	-25.794	152.586
W02-MB5	wild		*M.integrifolia*	Australia	MtBauple3	-24.794	153.586
W04-MB04	wild		*M.integrifolia*	Australia	MtBauple4	-25.827	152.588
W05-MB05	wild		*M.integrifolia*	Australia	MtBauple5	-25.807	152.585
W06-MCk03	wild		*M.integrifolia*	Australia	MaryCreek	-26.253	152.550

W06-MCk06	wild		*M.integrifolia*	Australia	MaryCreek	-26.253	152.550
W08-Mo04	wild		*M.integrifolia*	Australia	Mooloo1	-26.309	152.611
W11-Am6	wild		*M.integrifolia*	Australia	AmamoorCk2	-26.359	152.646
W15-Vi04	wild		*M.integrifolia*	Australia	Villeneuve	-26.973	152.648
W17-UC2	wild		*M.integrifolia*	Australia	UpperCaboolture	-26.987	152.844
W17-UC4	wild		*M.integrifolia*	Australia	UpperCaboolture	-26.987	152.844
W17-UC6	wild		*M.integrifolia*	Australia	UpperCaboolture	-26.987	152.844
W20-Sa4	wild		*M.integrifolia*	Australia	Samford2	-27.342	152.818
W8b-Mo10	wild		*M.integrifolia*	Australia	Mooloo1	-26.309	152.611
W8b-Mo11	wild		*M.integrifolia*	Australia	Mooloo1	-26.309	152.611
W8b-Mo14	wild		*M.integrifolia*	Australia	Mooloo1	-26.309	152.611
W9b-Mo07	wild		*M.integrifolia*	Australia	Mooloo2	-26.325	152.620
W-Am279	wild		*M.integrifolia*	Australia	AmamoorLTM	-26.325	152.562
W-Lan-02	wild		*M.integrifolia*	Australia	LangshawLTM	-26.317	152.562
W-Lan-03	wild		*Floydia*	Australia	LangshawLTM	-25.317	153.562
W-Mck02	wild		*M.integrifolia*	Australia	MaryCreek	-26.253	152.550
W-Mck03	wild		*M.integrifolia*	Australia	MaryCreek	-25.253	153.550
M36			*M.ternifolia*	Hawaii			
M17	PROBERT2	HAES865	*M.tetraphylla*	Hawaii			
M37			*Hicksbeachia*	Hawaii			
M35			*Lasjiahildebrandii*	Hawaii			

Supplementary Table 22. List of 60 Hawaiian cultivars and wild accessions used in domestication analysis

sample ID	Source	Group
M1	Hawaii	cultivars
M2	Hawaii	cultivars
M3	Hawaii	cultivars
M4	Hawaii	cultivars
M5	Hawaii	cultivars
M6	Hawaii	cultivars
M7	Hawaii	cultivars
M9	Hawaii	cultivars
M23	Hawaii	cultivars
M24	Hawaii	cultivars
M25	Hawaii	cultivars
M26	Hawaii	cultivars
M27	Hawaii	cultivars
M29	Hawaii	cultivars
M32	Hawaii	cultivars
M38	Hawaii	cultivars
M39	Hawaii	cultivars
M40	Hawaii	cultivars
M41	Hawaii	cultivars
M43	Hawaii	cultivars
M44	Hawaii	cultivars
M45	Hawaii	cultivars

M47	Hawaii	cultivars
M835	Hawaii	cultivars
s1001-002	Australia	c1
s1001-003	Australia	c1
s1003-001	Australia	c1
s1008-005	Australia	c2
s1009-003	Australia	c2
s1009-004	Australia	c2
s1009-006	Australia	c2
s1020-001	Australia	c3
s1020-006	Australia	c3
s1053-005	Australia	c1
s1055-002	Australia	c2
s1055-006	Australia	c2
s1058-003	Australia	c2
s1076-003	Australia	c3
s1076-005A	Australia	c3
s1076-005B	Australia	c3
s1077-006A	Australia	c3
s1077-006B	Australia	c3
W01-MB1	Australia	c1
W02-MB3	Australia	c1
W02-MB5	Australia	c1
W04-MB04	Australia	c1
W05-MB05	Australia	c1
W06-MCk03	Australia	c2
W06-MCk06	Australia	c2
W08-Mo04	Australia	c2
W11-Am6	Australia	c2
W20-Sa4	Australia	c3
W8b-Mo10	Australia	c2
W8b-Mo11	Australia	c2
W8b-Mo14	Australia	c2
W9b-Mo07	Australia	c2
W-Am279	Australia	c2
W-Lan-02	Australia	c2
W-Mck02	Australia	c2
W-Mck03	Australia	c2

Supplemental Figures

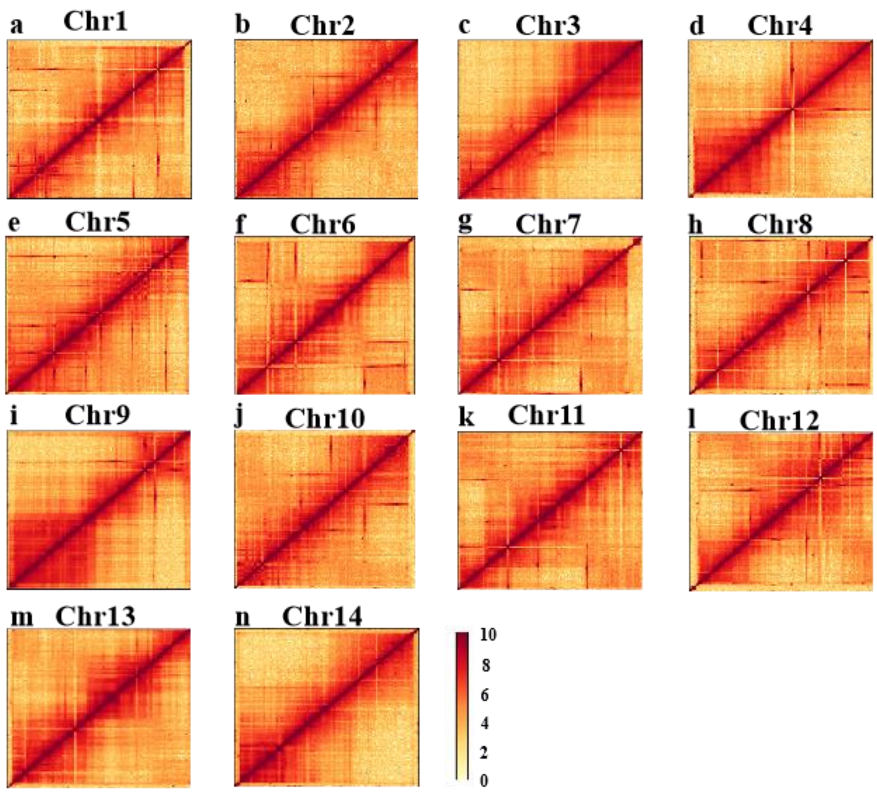

Supplementary Figure 1. Hi-C assistant mapping of 14 chromosomes of macadamia. Hi-C heatmaps are shown at 150 kb resolution. **(a)** Hi-C heatmaps of Chromosome 1. **(b)** Hi-C heatmaps of Chromosome 2. **(c)** Hi-C heatmaps of Chromosome 3. **(d)** Hi-C heatmaps of Chromosome 4. **(e)** Hi-C heatmaps of Chromosome 5. **(f)** Hi-C heatmaps of Chromosome 6. **(g)** Hi-C heatmaps of Chromosome 7. **(h)** Hi-C heatmaps of Chromosome 8. **(i)** Hi-C heatmaps of Chromosome 9. **(j)** Hi-C heatmaps of Chromosome 10. **(k)** Hi-C heatmaps of Chromosome 11. **(l)** Hi-C heatmaps of Chromosome 12. **(m)** Hi-C heatmaps of Chromosome 13. **(n)** Hi-C heatmaps of Chromosome 14.

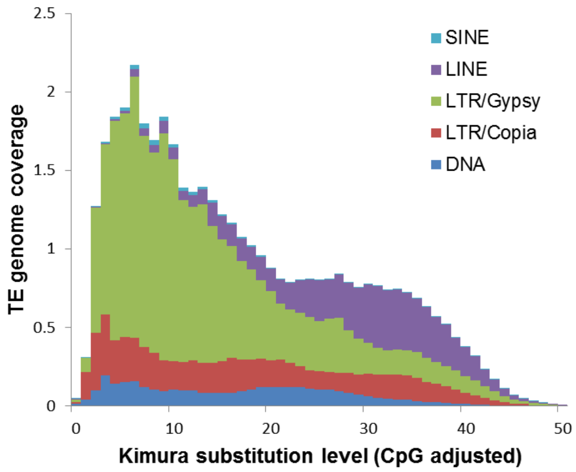

Supplementary Figure 2. Kimura distance analysis.

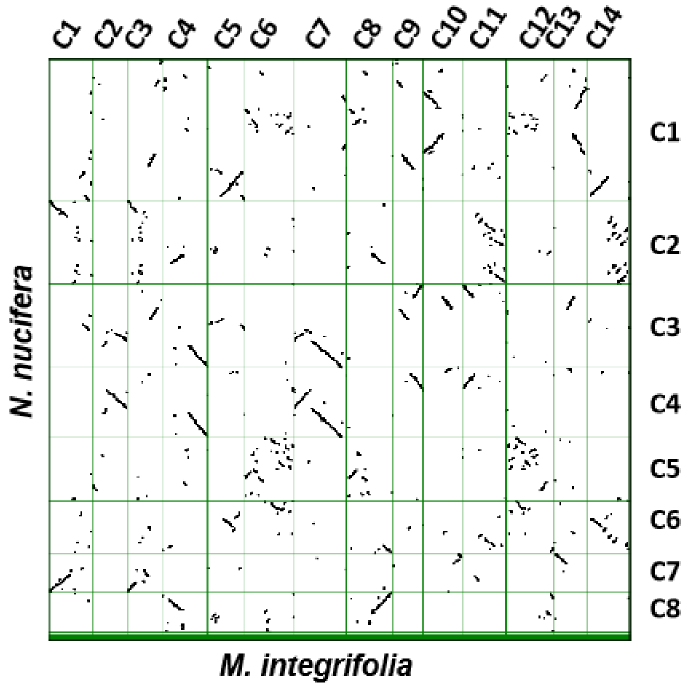

Supplementary Figure 3. The synteny blocks between *M. integrifolia* and *N. nucifera*.

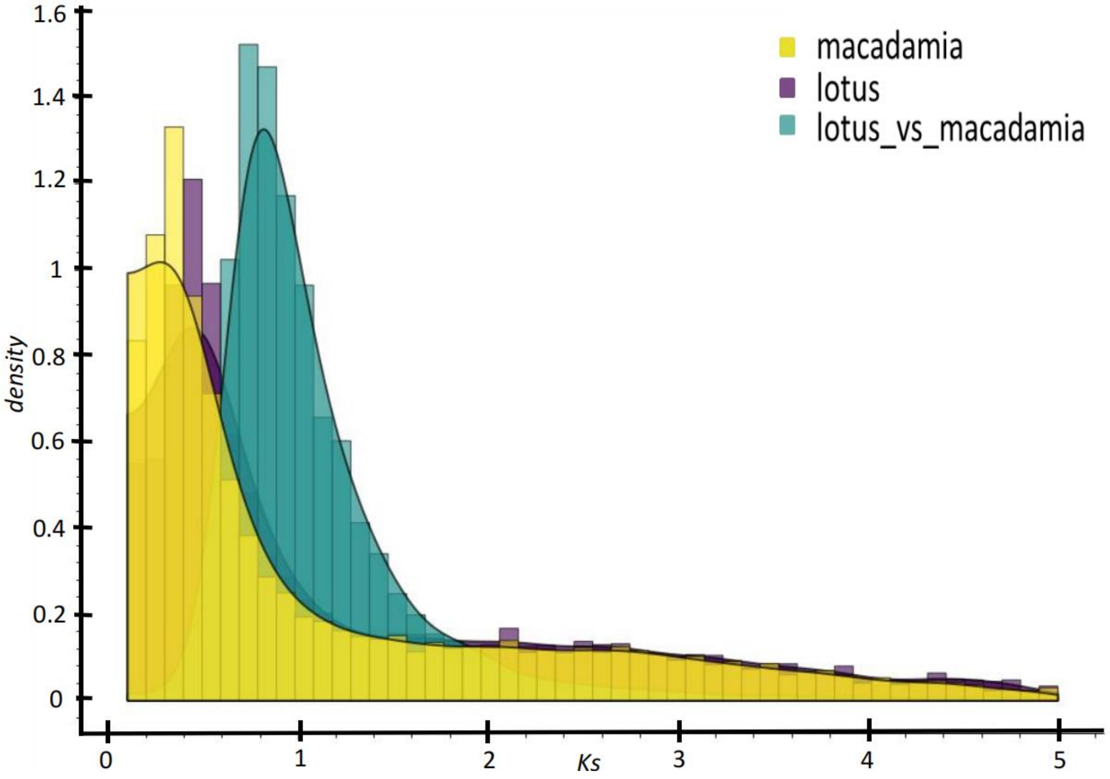

Supplementary Figure 4. Ks distributions of syntenic blocks for *M. integrifolia* and paralogs and orthologs with *N. nucifera* are shown by coloured lines, as indicated.

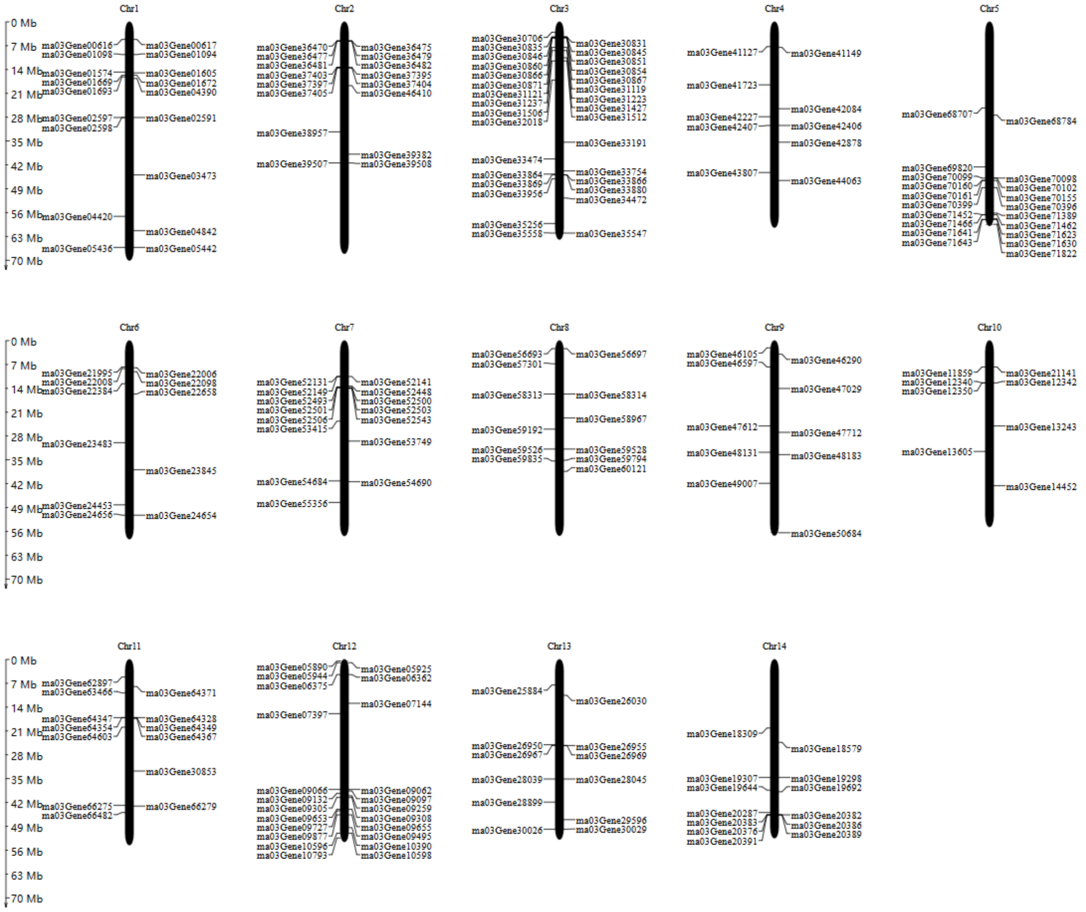

Supplementary Figure 5. Distribution of species-specific genes on fourteen chromosomes.

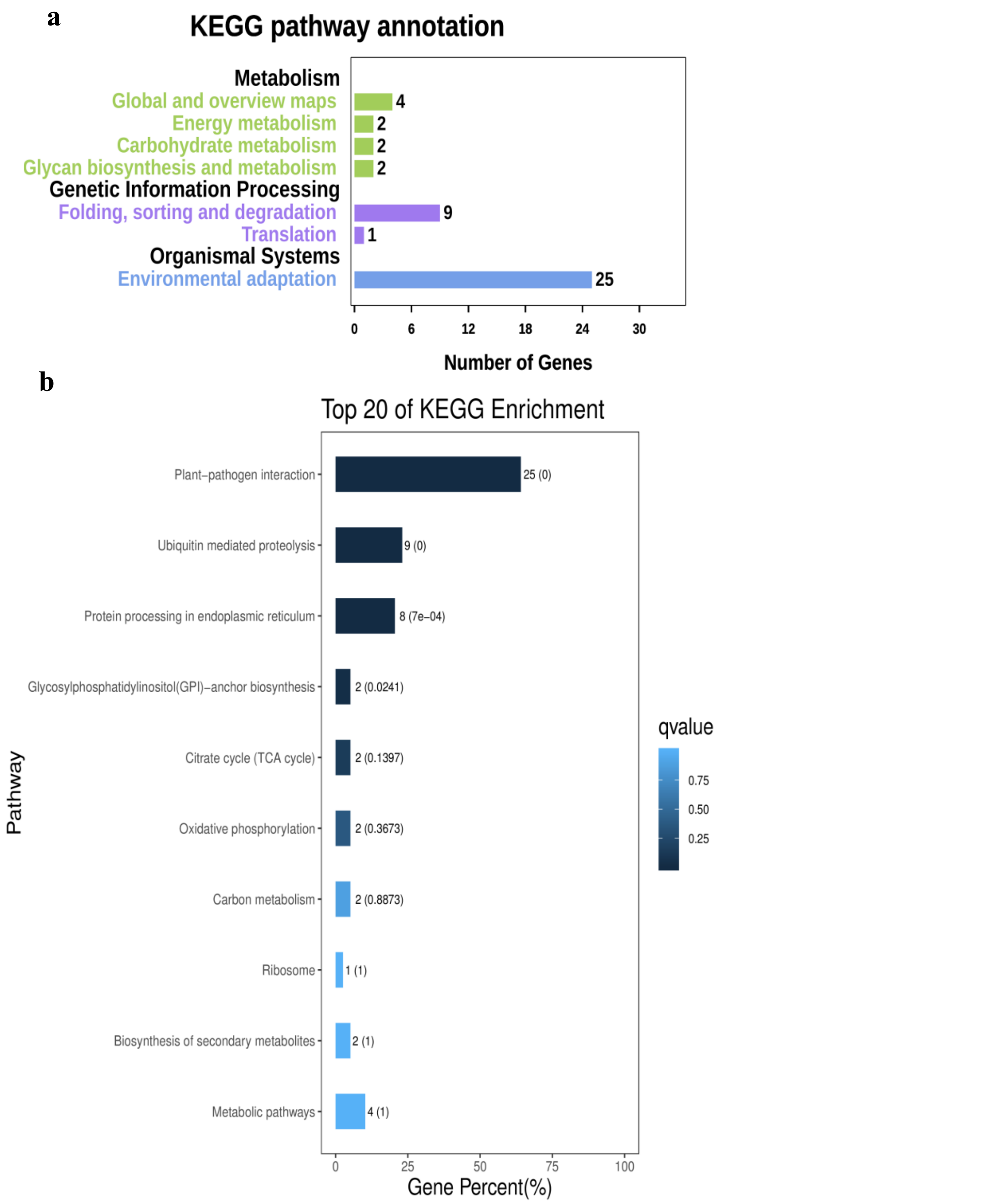

Supplementary Figure 6. Pathway enrichment of species-specific genes of macadamia. (a) Annotation of eniched KEGG pathways. **(b)** Top 20 of eniched KEGG pathways.

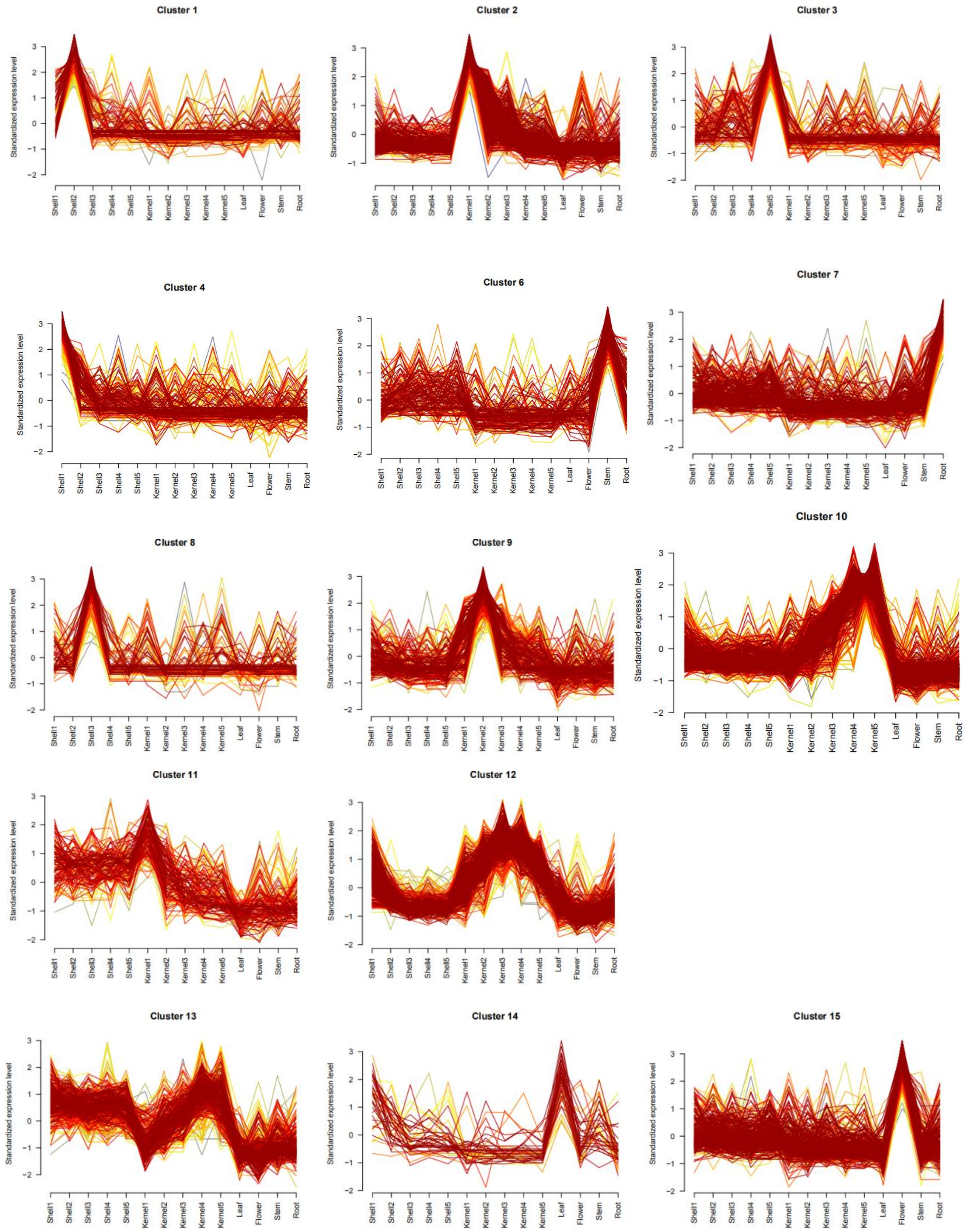

Supplementary Figure 7. Mfuzz clustering of differentially expressed transcripts in shells.

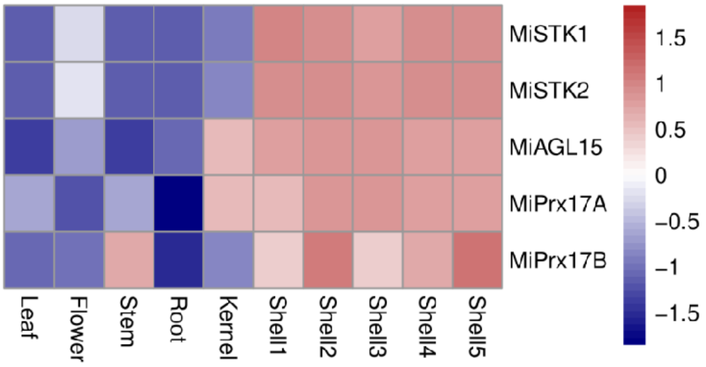

Supplementary Figure 8. Expression of *STKs* and *Prx17s* in different tissues of macadamia. Source data are provided as a Source Data file.

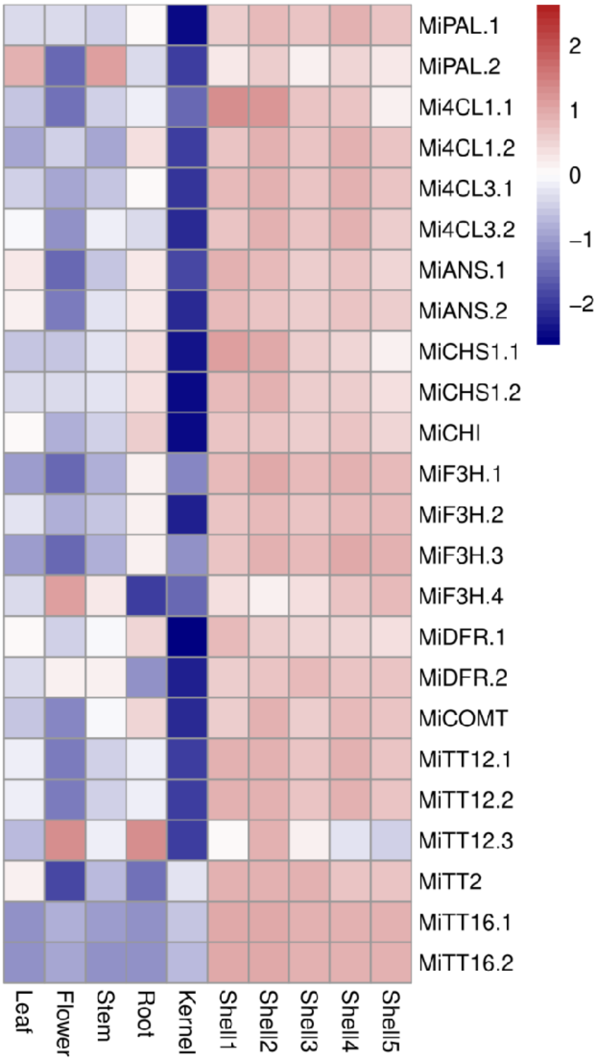

Supplementary Figure 9. Expression of phenylpropanoid biosynthesis related genes in different tissues of macadamia. Source data are provided as a Source Data file.

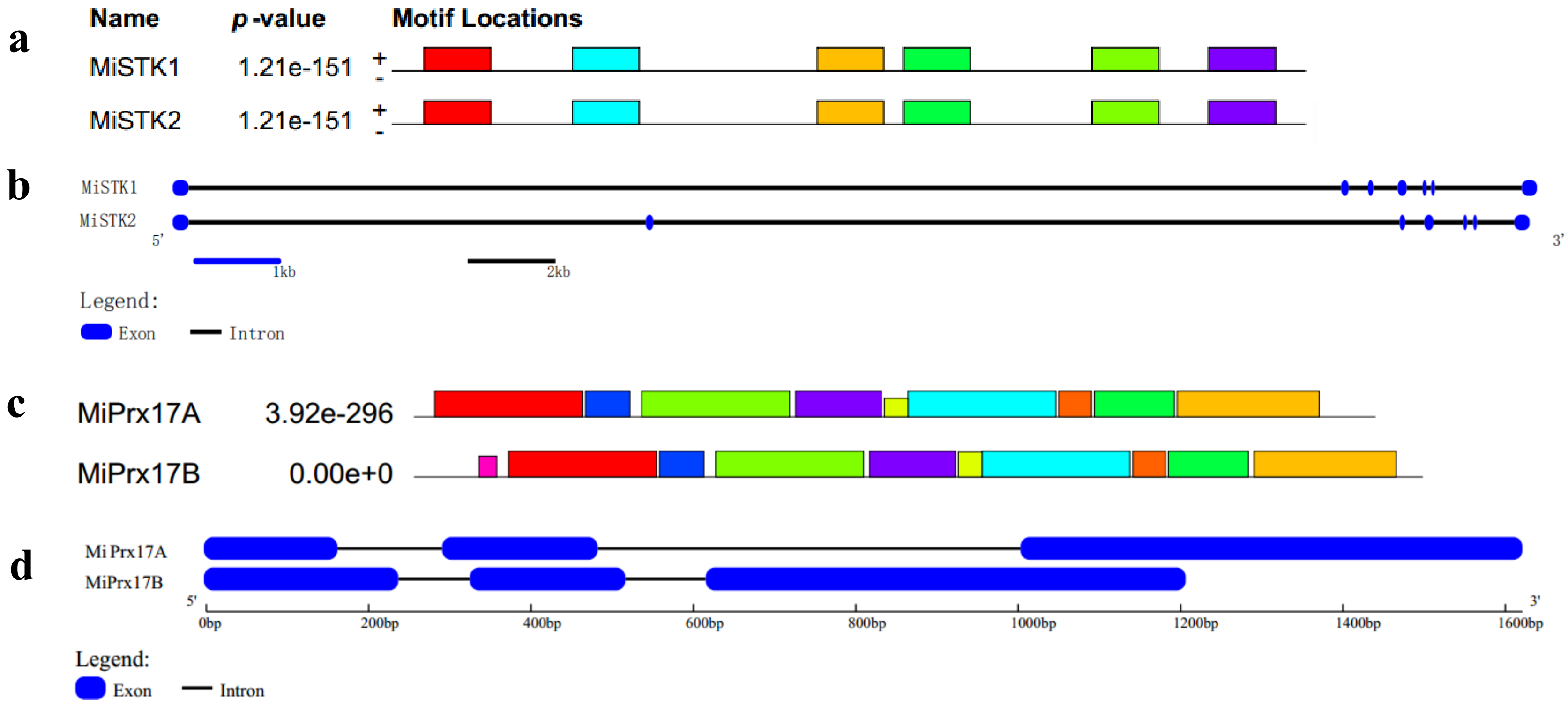

Supplementary Figure 10. Comparison of *STK* and *Prx17* genes in macadamia. (a) Protein motiff of three *STK* genes of macadamia. **(b)** gene structure of three *STK* genes of macadamia. **(c)** Protein motiff of three *Prx* genes of macadamia. **(d)** gene structure of three *Prx* genes of macadamia.

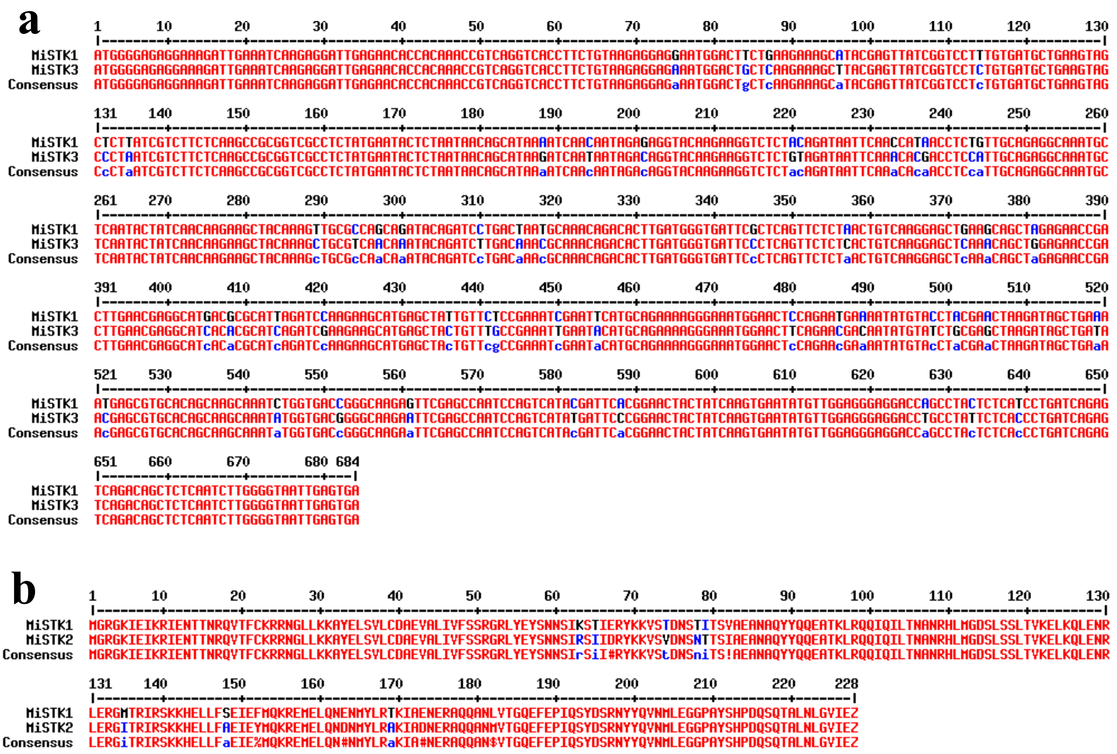

Supplementary Figure 11. Coding and protein sequencing of four *MaSTK* genes

(a) multiple coding sequence alignment. **(b)** multiple protein sequence alignment.

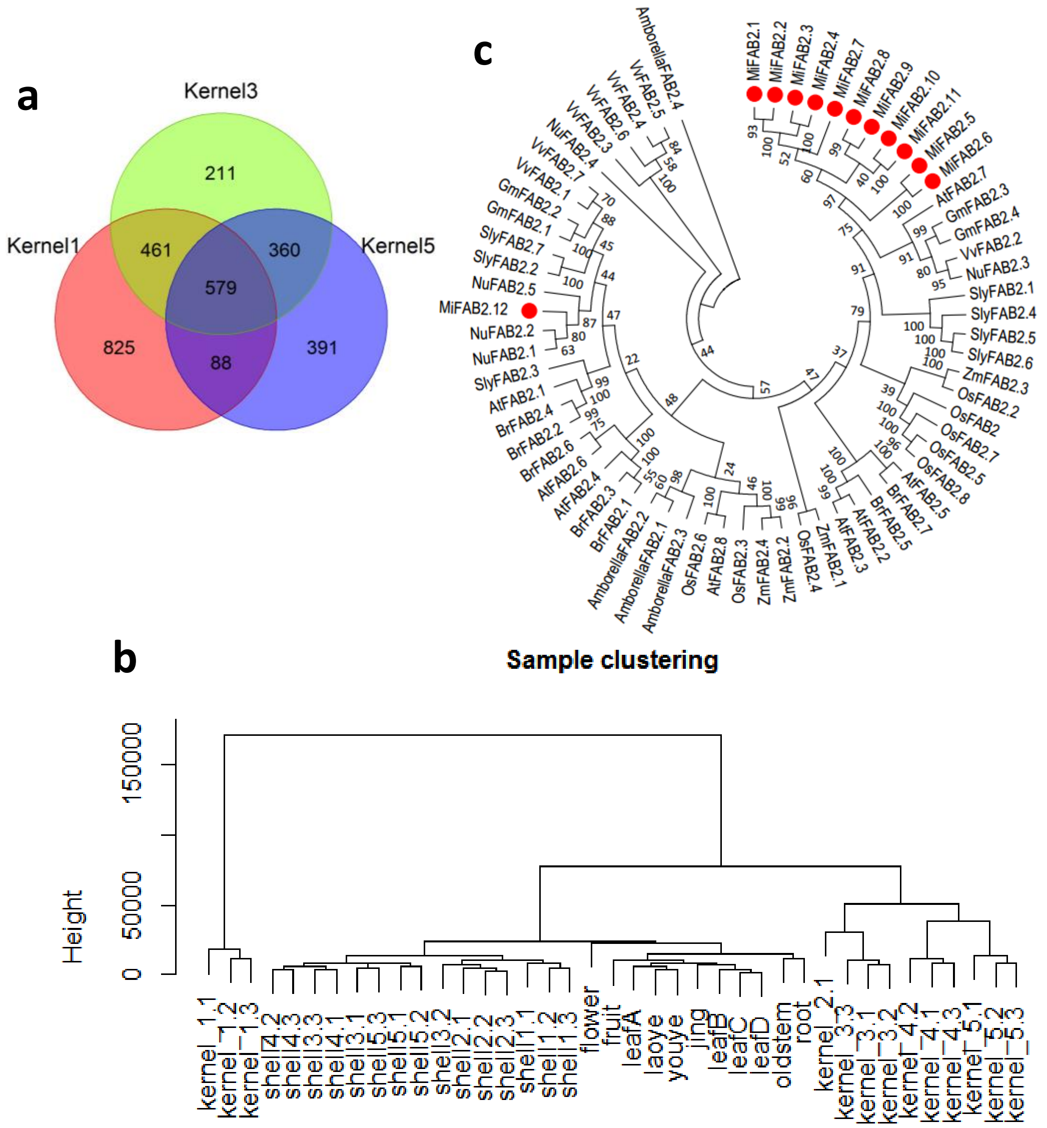

Supplementary Figure 12. Deferentially expressed genes at three stages of kernel development. (a) Venn plot of up expression genes in three stages of kernels. **(b)** Sample clustering of different tissues**. (c)** Phylogeny of the expanded *FAB* gene family in *M. integrifolia.*

a

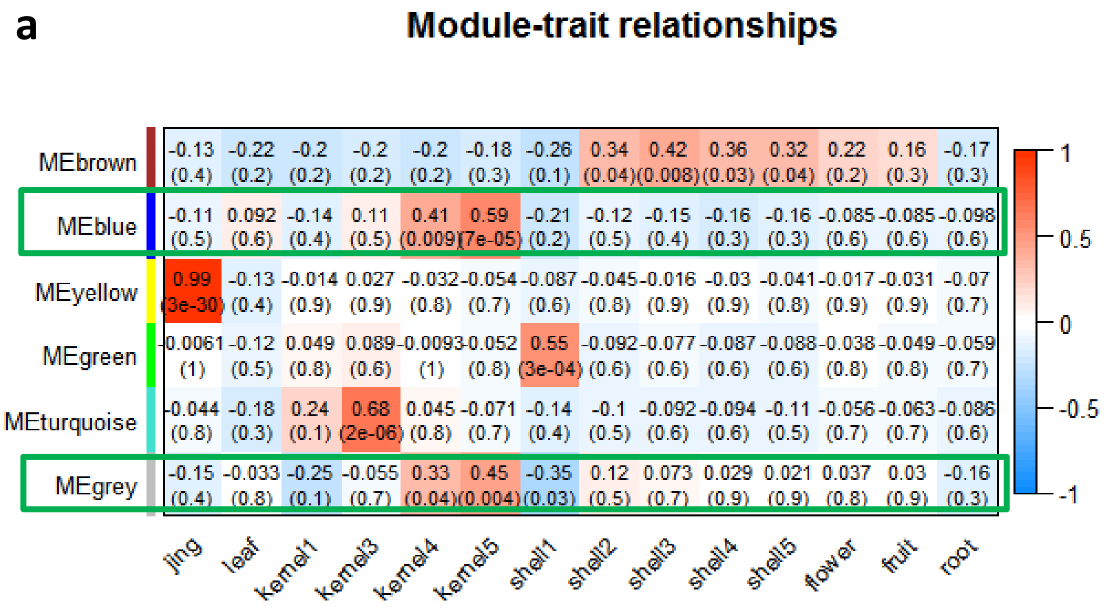

b

Module	MEblue	MEbrown	MEgreen	MEgrey	MEturquoise	MEyellow
Genes number	591	122	70	488	1072	110

Supplementary Figure 13. WGCNA analysis of kernel transcriptome. (a) Module-trait relationships. Green rectangle indicate kernel development related module. **(b)** Gene numbers in every modules

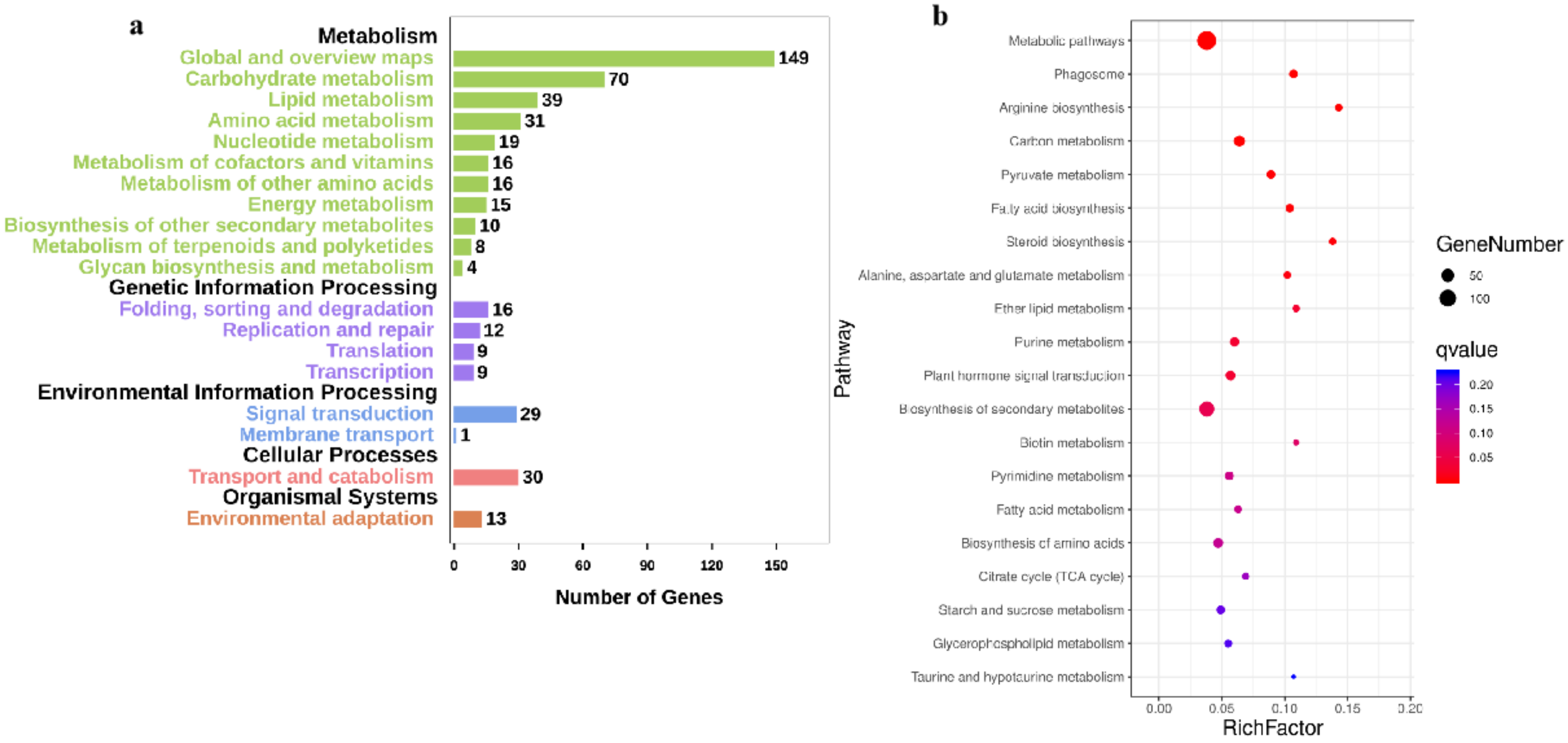

Supplementary Figure 14. Pathway enrichment of high expressed and correlated genes in kernels. (a) Annotation of eniched KEGG pathways. **(b)** Top 20 of eniched KEGG pathways

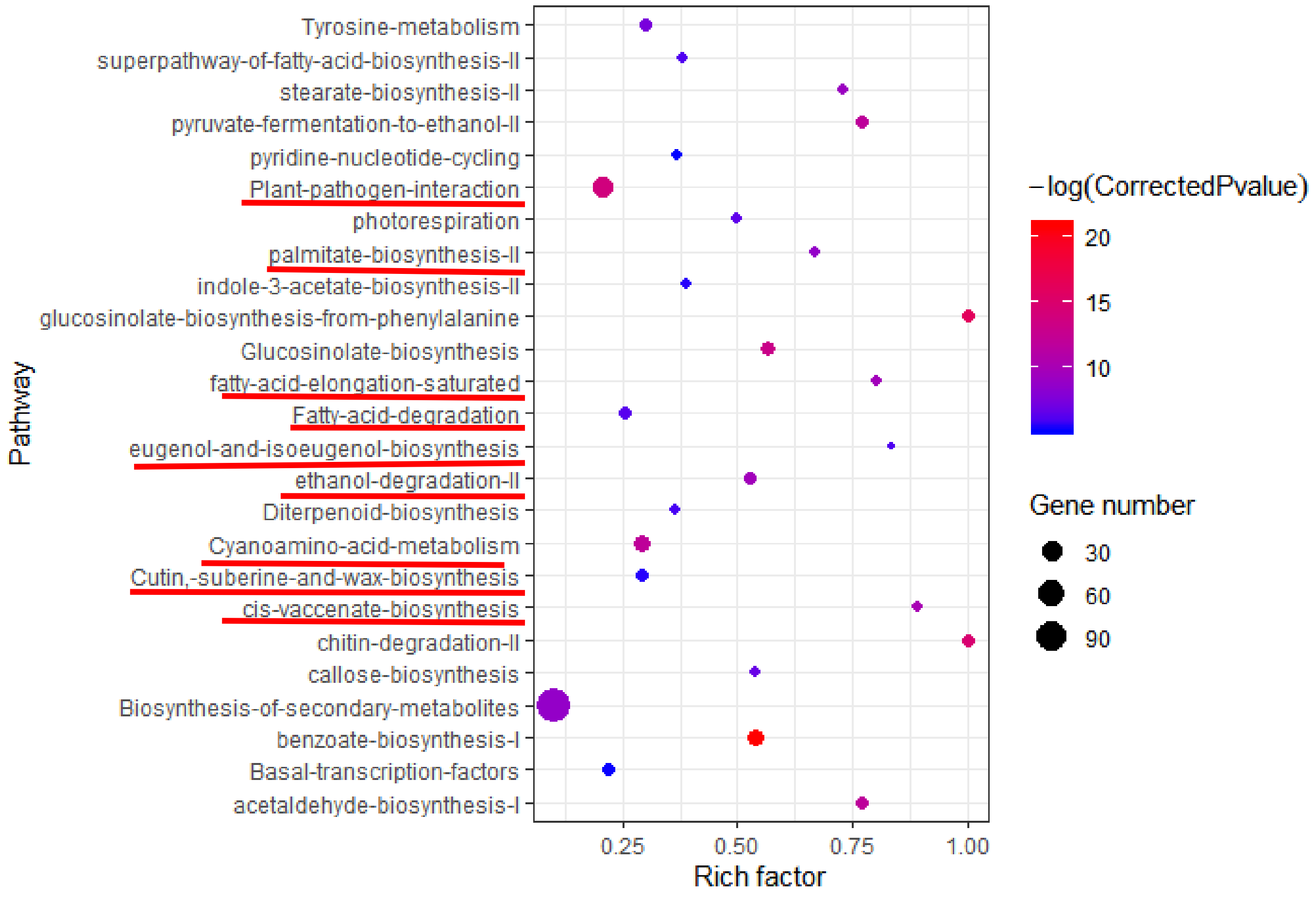

Supplementary Figure 15. Pathway enrichment of expanded genes in *M. integrifolia* genome. Underline pathways indicate relationship with fatty-acid and oil biosynthesis.

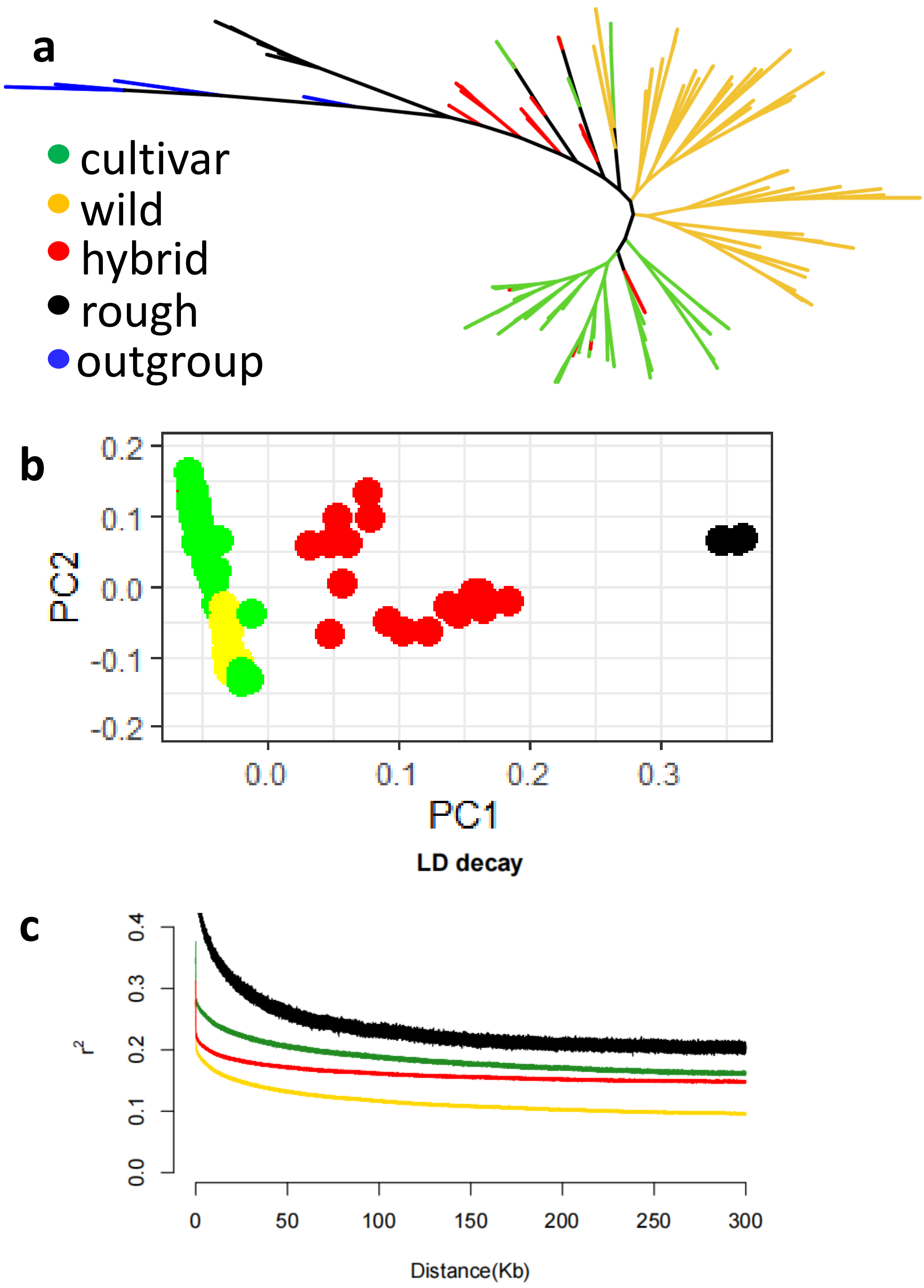

Supplementary Figure 16. Phylogenetic relationships resequenced macadamia 112 individuals. **(a)** The maximum likelihood tree and **(b)** Principal Component Analysis (PCA) of 108 re-sequenced tea individuals with horizontal and vertical axes explaining 18.7% and 7.7% of the variance, respectively. **(c)** Decay of linkage disequilibrium (LD), measured by r^2, in each group. Cultivar represent cultivars of *M. integrifolia* from Hawaii; hybrid represent corss of *M. integrifolia* and *M. tetraphylla*; wild represent wild lines of *M. integrifolia* from Australia; rough represent cultivars of *M. tetraphylla*.

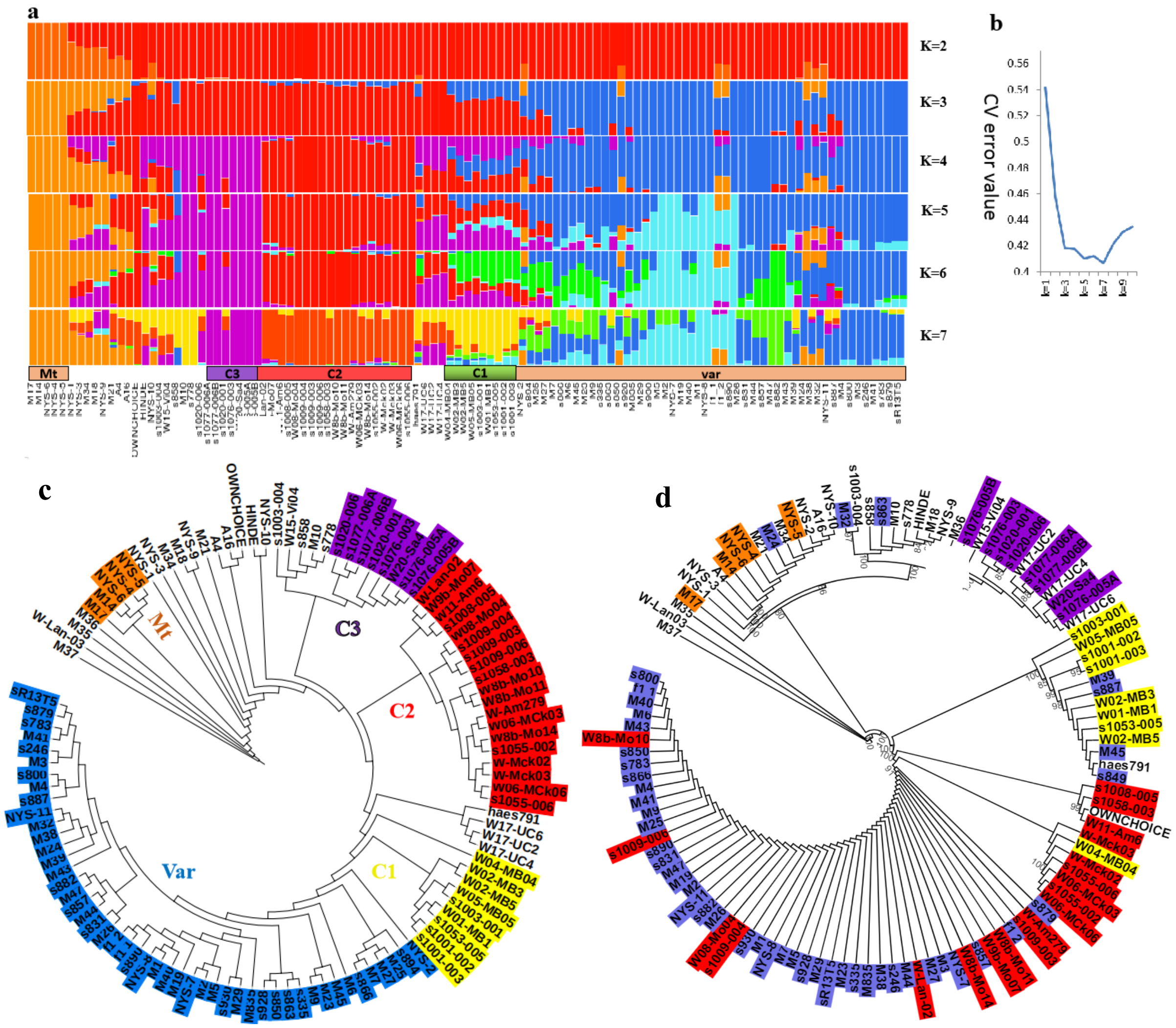
a
K=2
K=3
K=4
K=5
K=6
K=7
Mt
C3
C2
C1
var
b
CV error value
0.56
0.54
0.52
0.5
0.48
0.46
0.44
0.42
0.4
c
Mt
C3
C2
C1
Var
d

Supplementary Figure 17. Phylogenetic relationships and population structures of macadamia individuals. (a) Population structure of the 112 macadamia accessions, including varities of Hawai'ian (cultivars), wild from Australia (C1, C2 and C3) and accessions of *M. tetraphylla* (Mt). **(b)** K values based on admixture analysis. **(c)** Maximum likelihood tree based on nuclear genome-wide SNPs. **(d)** Maximum likelihood tree based on chloroplast genome-wide SNPs.

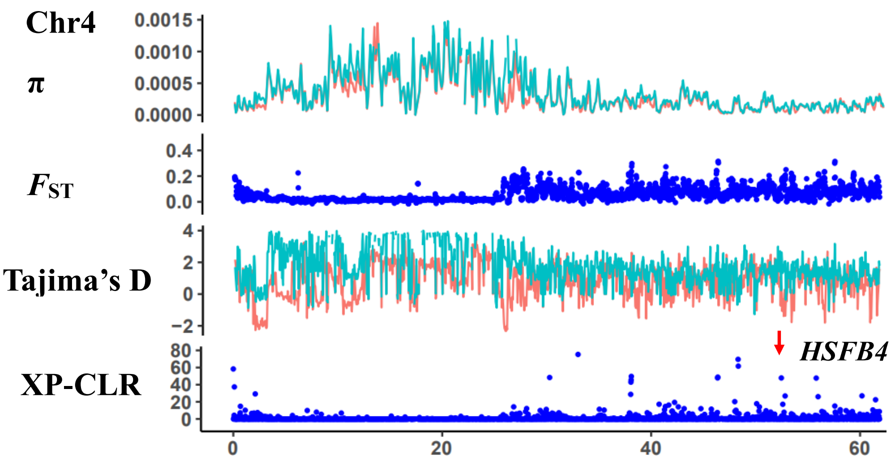

Supplementary Figure 18. Signatures of selected signals in Chromosome 4 of *M. integrifolia* genome. The upmost dotplot is nucleotide diversity (π) values, red indicate varieties and green line indicate wild group;the second lay is fixation index (F_{ST}) between the wild and cultivated macadamia accessions; the third lay is Tajima's D values, red indicate varieties and green line indicate wild group. the bottom is genome-wide distribution of selective-sweep signals identified based on the cross-population composite likelihood ratio test (XP-CLR); *HSFB4* heat stress transcription factor.

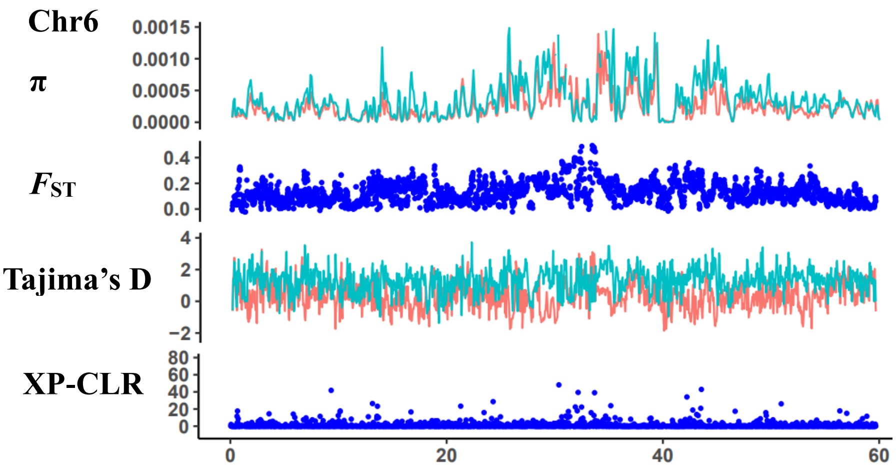

Supplementary Figure 19. Signatures of selected signals in Chromosome 6 of *M. integrifolia* genome. The upmost dotplot is nucleotide diversity (π) values, red indicate varieties and green line indicate wild group;the second lay is fixation index (F_{ST}) between the wild and cultivated macadamia accessions; the third lay is Tajima's D values, red indicate varieties and green line indicate wild group. the bottom is genome-wide distribution of selective-sweep signals identified based on the cross-population composite likelihood ratio test (XP-CLR).

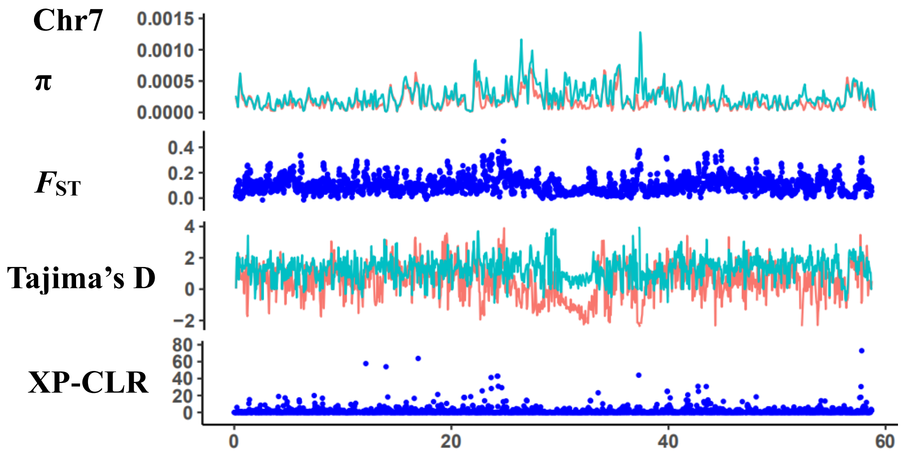

Supplementary Figure 20. Signatures of selected signals in Chromosome 7 of *M. integrifolia* genome. The upmost dotplot is nucleotide diversity (π) values, red indicate varieties and green line indicate wild group;the second lay is fixation index (F_{ST}) between the wild and cultivated macadamia accessions; the third lay is Tajima's D values, red indicate varieties and green line indicate wild group. the bottom is genome-wide distribution of selective-sweep signals identified based on the cross-population composite likelihood ratio test (XP-CLR).

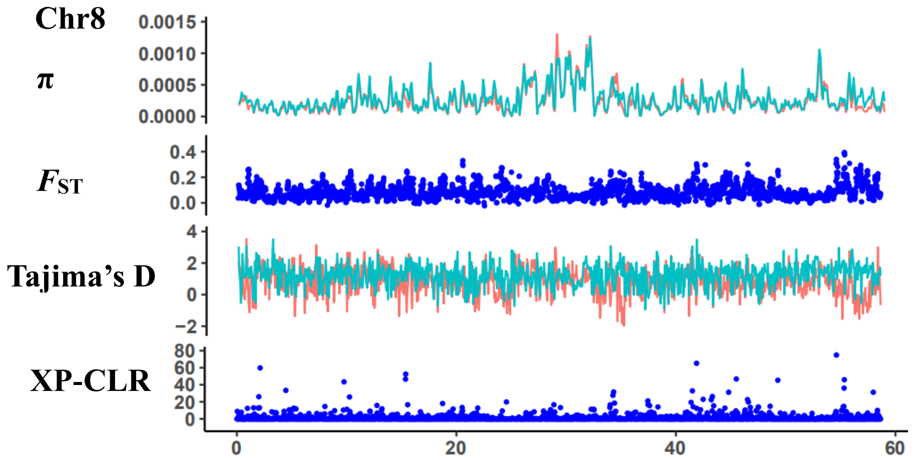

Supplementary Figure 21. Signatures of selected signals in Chromosome 8 of *M. integrifolia* genome. The upmost dotplot is nucleotide diversity (π) values, red indicate varieties and green line indicate wild group;the second lay is fixation index (F_{ST}) between the wild and cultivated macadamia accessions; the third lay is Tajima's D values, red indicate varieties and green line indicate wild group. the bottom is genome-wide distribution of selective-sweep signals identified based on the cross-population composite likelihood ratio test (XP-CLR).

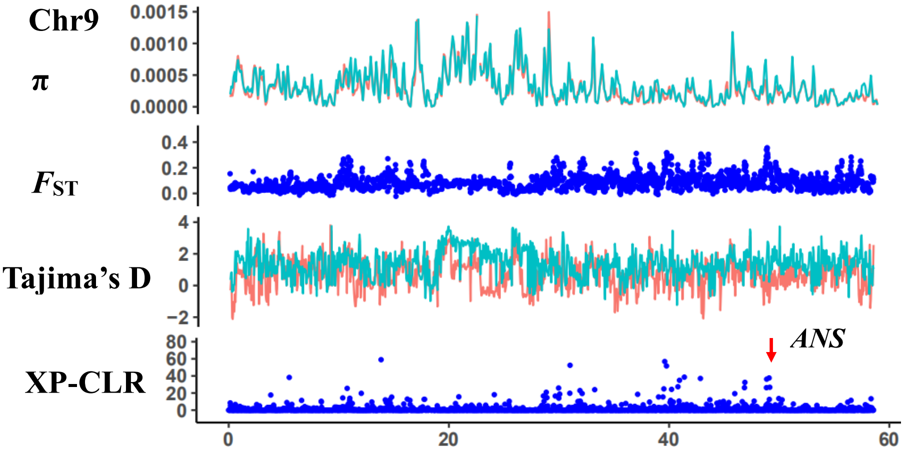

Supplementary Figure 22. Signatures of selected signals in Chromosome 9 of *M. integrifolia* genome. The upmost dotplot is nucleotide diversity (π) values, red indicate varieties and green line indicate wild group;the second lay is fixation index (F_{ST}) between the wild and cultivated macadamia accessions; the third lay is Tajima's D values, red indicate varieties and green line indicate wild group. the bottom is genome-wide distribution of selective-sweep signals identified based on the cross-population composite likelihood ratio test (XP-CLR).

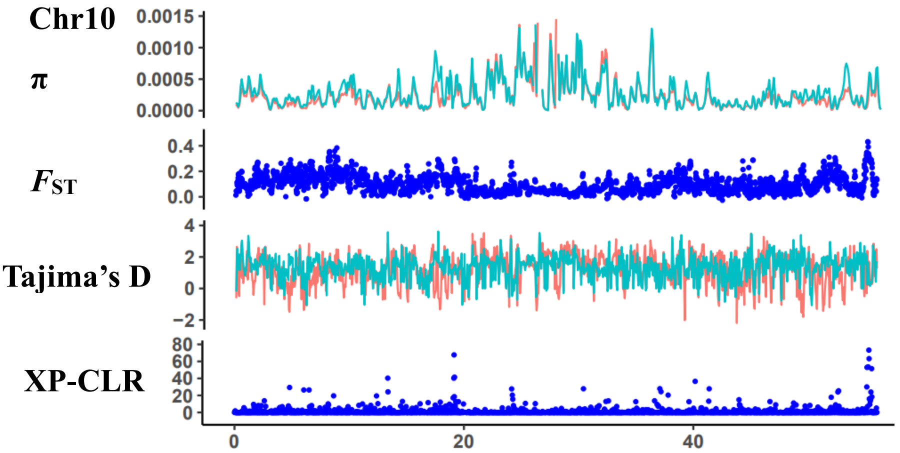

Supplementary Figure 23. Signatures of selected signals in Chromosome 10 of *M. integrifolia* genome. The upmost dotplot is nucleotide diversity (π) values, red indicate varieties and green line indicate wild group;the second lay is fixation index (F_{ST}) between the wild and cultivated macadamia accessions; the third lay is Tajima's D values, red indicate varieties and green line indicate wild group. the bottom is genome-wide distribution of selective-sweep signals identified based on the cross-population composite likelihood ratio test (XP-CLR).

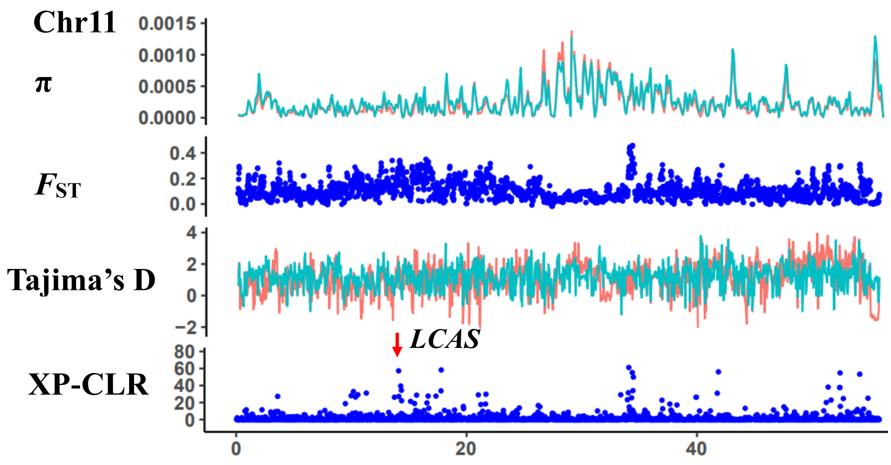

Supplementary Figure 24. Signatures of selected signals in Chromosome 11 of *M. integrifolia* genome. The upmost dotplot is nucleotide diversity (π) values, red indicate varieties and green line indicate wild group;the second lay is fixation index (F_{ST}) between the wild and cultivated macadamia accessions; the third lay is Tajima's D values, red indicate varieties and green line indicate wild group. the bottom is genome-wide distribution of selective-sweep signals identified based on the cross-population composite likelihood ratio test (XP-CLR); *LCAS* Long chain acyl-CoA synthetase.

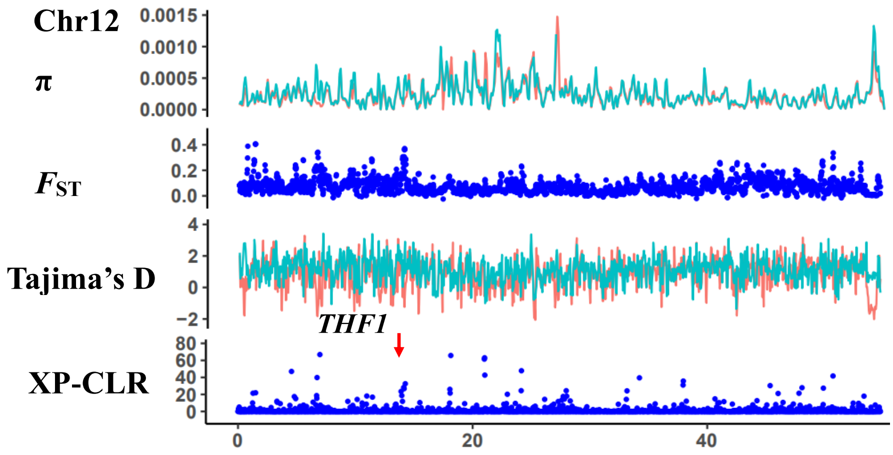

Supplementary Figure 25. Signatures of selected signals in Chromosome 12 of *M. integrifolia* genome. The upmost dotplot is nucleotide diversity (π) values, red indicate varieties and green line indicate wild group;the second lay is fixation index (F_{ST}) between the wild and cultivated macadamia accessions; the third lay is Tajima's D values, red indicate varieties and green line indicate wild group. the bottom is genome-wide distribution of selective-sweep signals identified based on the cross-population composite likelihood ratio test (XP-CLR); *THF*, THYLAKOID FORMATION Protein.

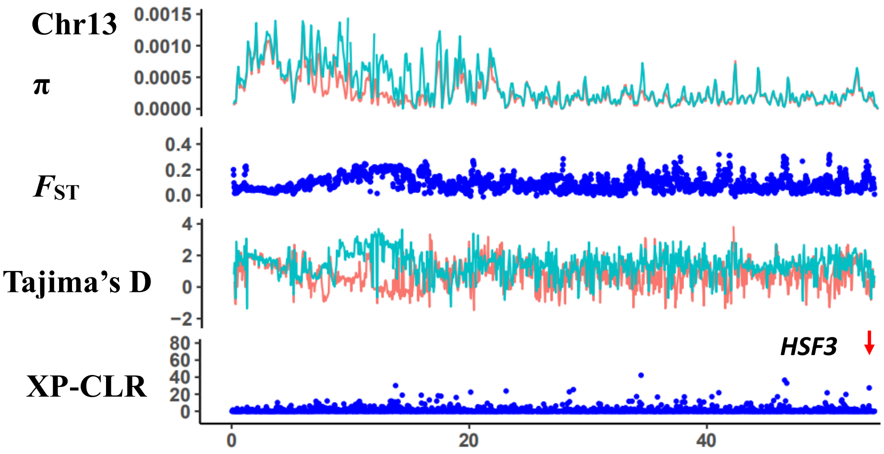

Supplementary Figure 26. Signatures of selected signals in Chromosome 13 of *M. integrifolia* genome. The upmost dotplot is nucleotide diversity (π) values, red indicate varieties and green line indicate wild group;the second lay is fixation index (F_{ST}) between the wild and cultivated macadamia accessions; the third lay is Tajima's D values, red indicate varieties and green line indicate wild group. the bottom is genome-wide distribution of selective-sweep signals identified based on the cross-population composite likelihood ratio test (XP-CLR); *HSF3* heat stress transcription factor.

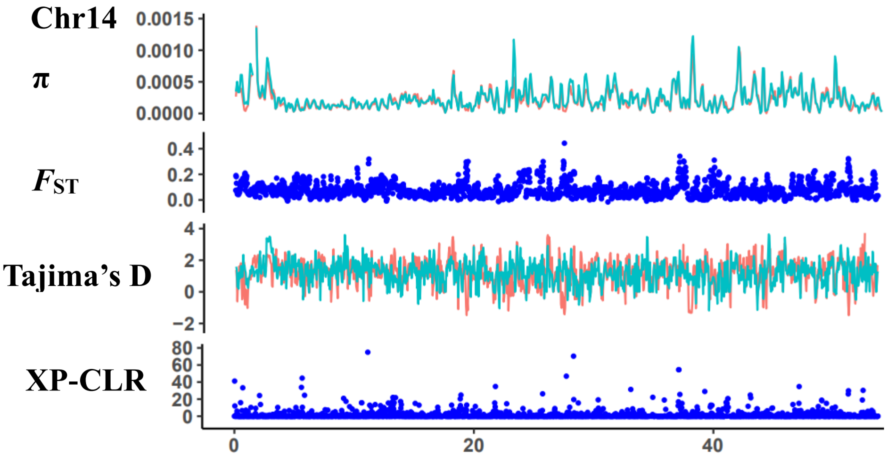

Supplementary Figure 27. Signatures of selected signals in Chromosome 14 of *M. integrifolia* genome. The upmost dotplot is nucleotide diversity (π) values, red indicate varieties and green line indicate wild group;the second lay is fixation index (F_{ST}) between the wild and cultivated macadamia accessions; the third lay is Tajima's D values, red indicate varieties and green line indicate wild group. the bottom is genome-wide distribution of selective-sweep signals identified based on the cross-population composite likelihood ratio test (XP-CLR).

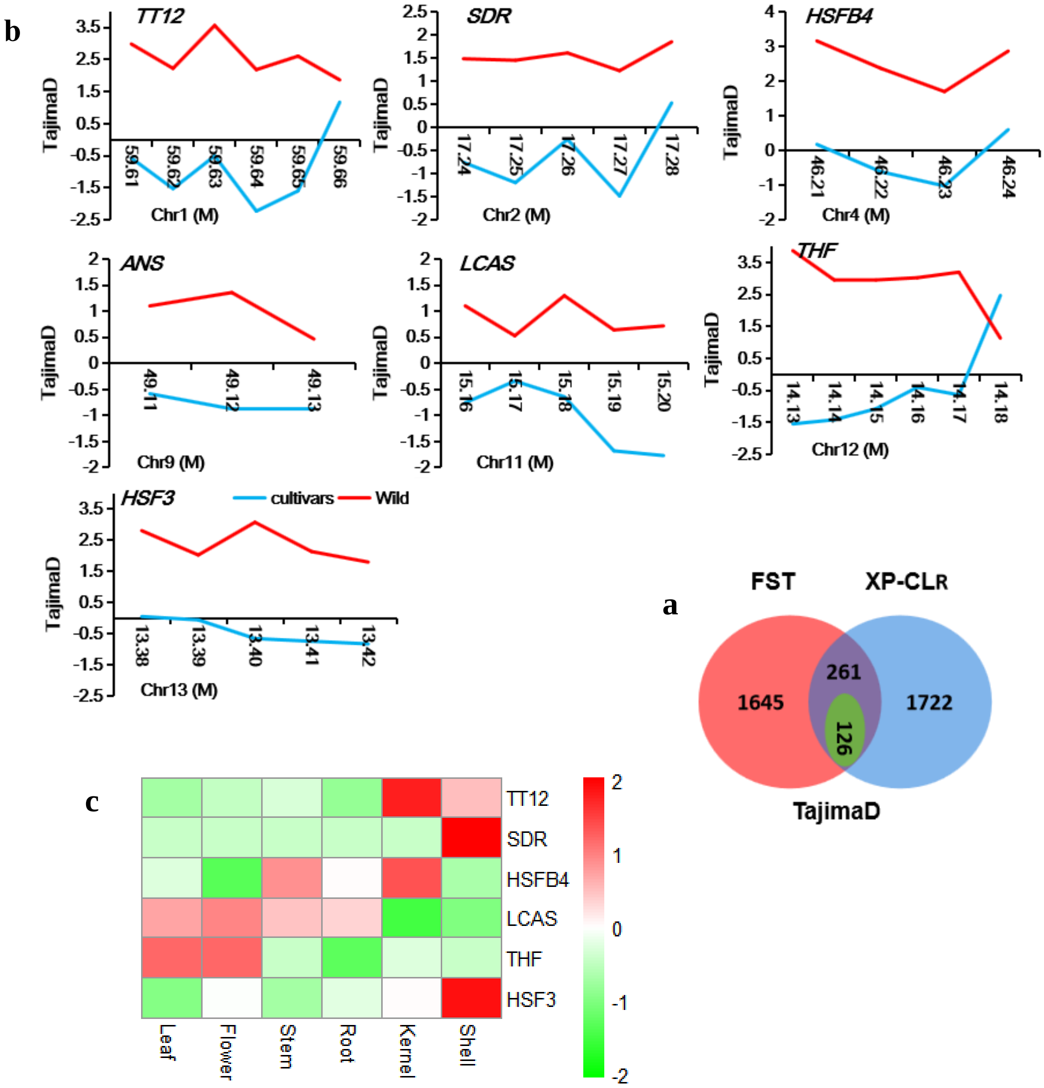

Supplementary Figure 28. Signatures of selected signals in *M. integrifolia*. (a) Top 5% of XP-CLR and F_{ST} blocks. Green represent blocks counts with negative Tajima's D in cultivatars and red represent positive TajimaD in wild. **(b)** Tajima's D of regions near selective-sweep genes between the wild and cultivated macadamia accessions. **(c)** Expression of z-score normalized selectively swept genes in six tissues of leaf, stem, flower, root, kernel and shell.

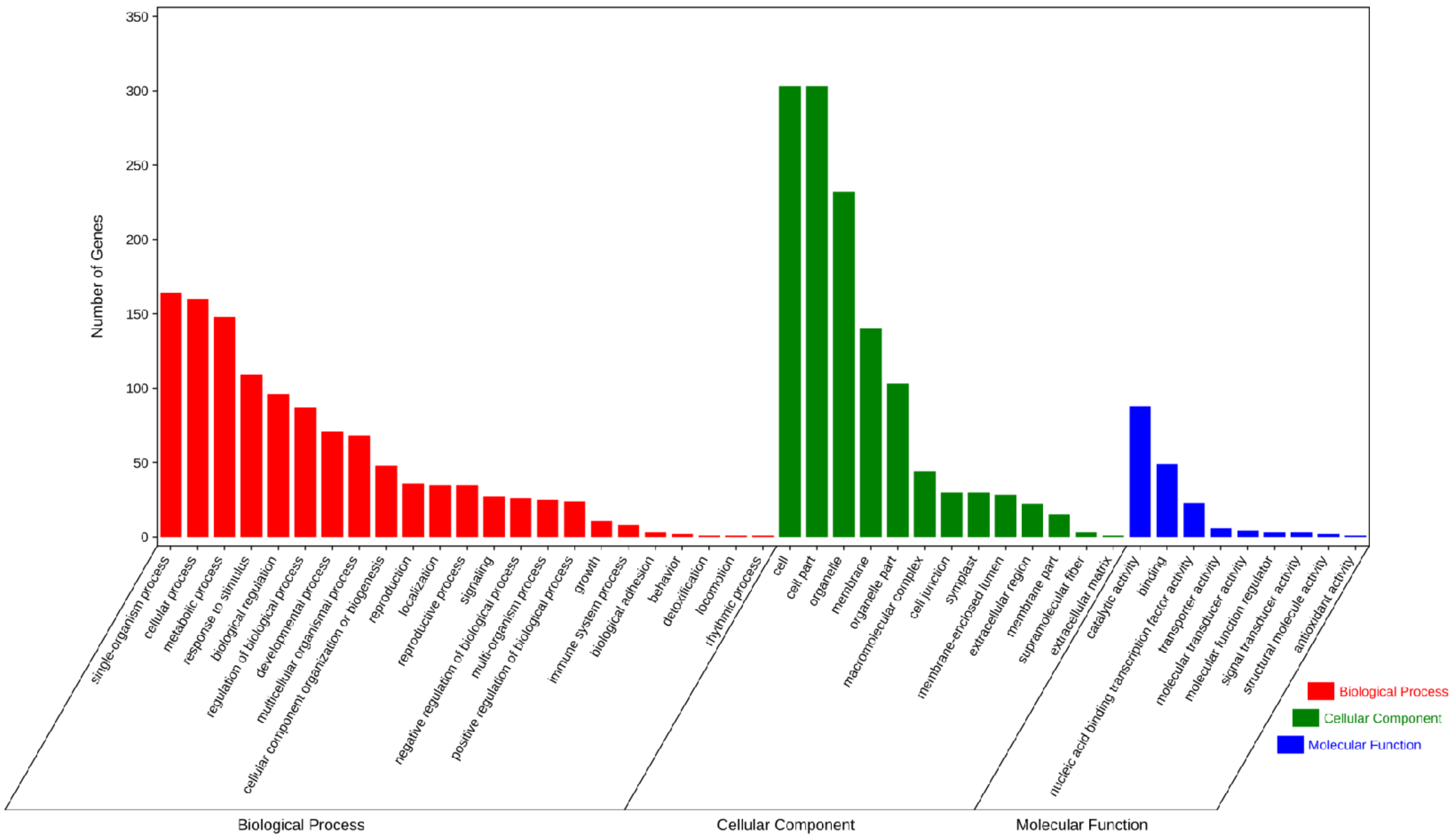

Supplementary Figure 29. GO enrichment of genes in swept signal blocks.

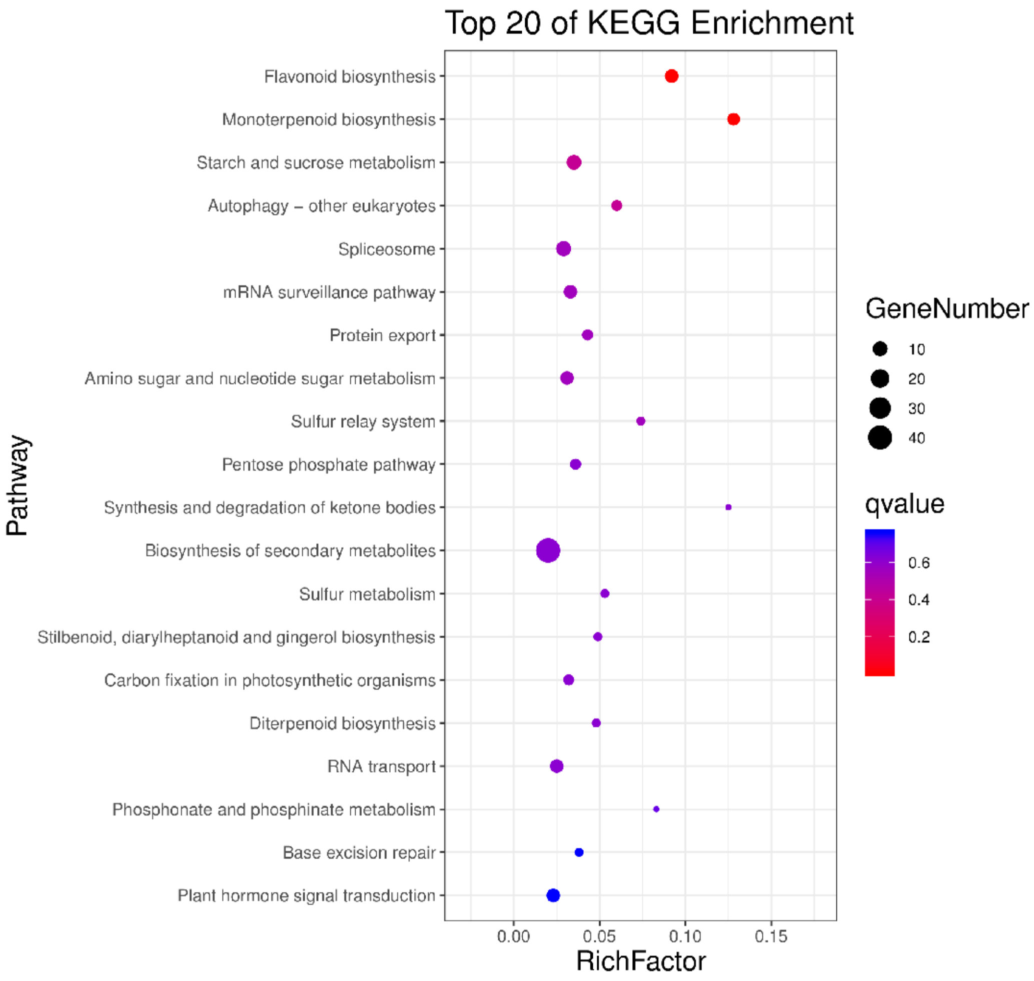

Supplementary Figure 30. KEGG enrichment of genes in swept signal blocks.

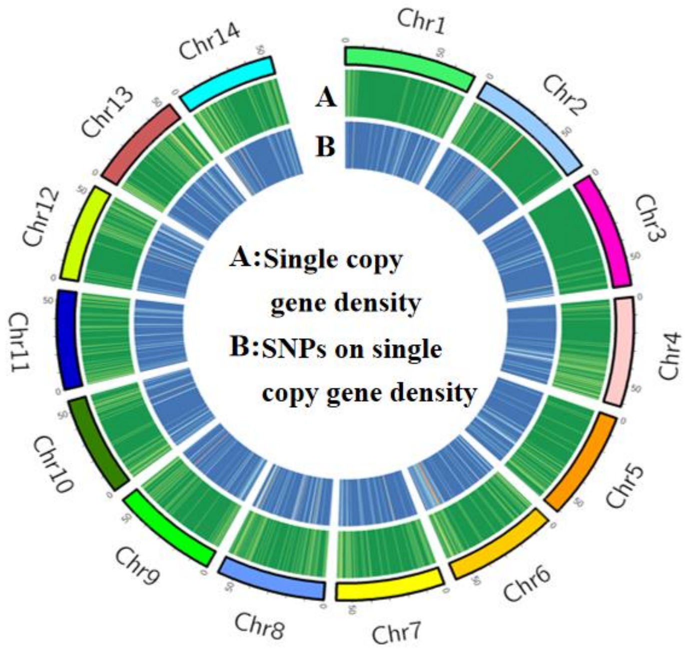

Supplementary Figure 31. Heatmap showing density of single copy gene and SNPs on single copy gene

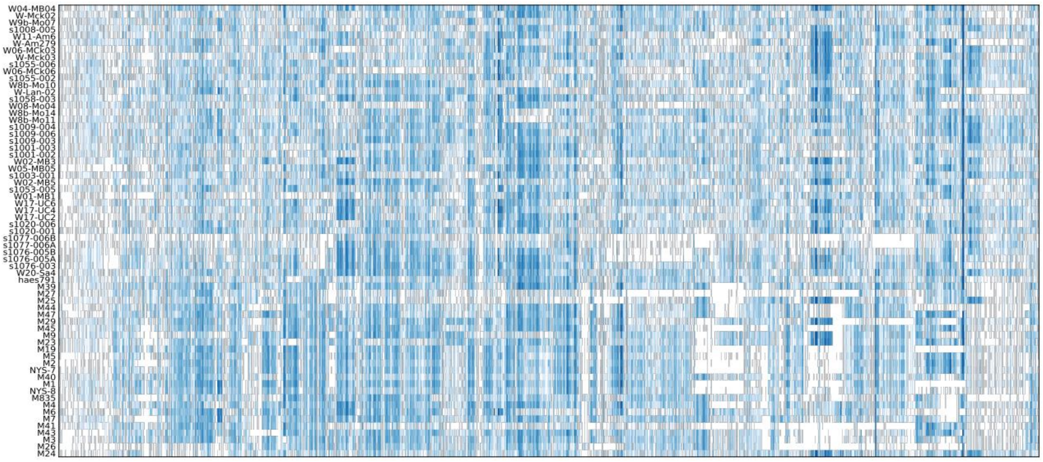

Chr 1

Supplementary Figure 32. Long tracks of homozygosity in Chromosome 1. Levels of heterozygosity were plotted for every 1000bp across 39 wild and 26 varieties accessions with low levels of admixture. A heatmap of heterozygosity is plotted where white indicates no heterozygosity and dark blue indicates high heterozygosity.

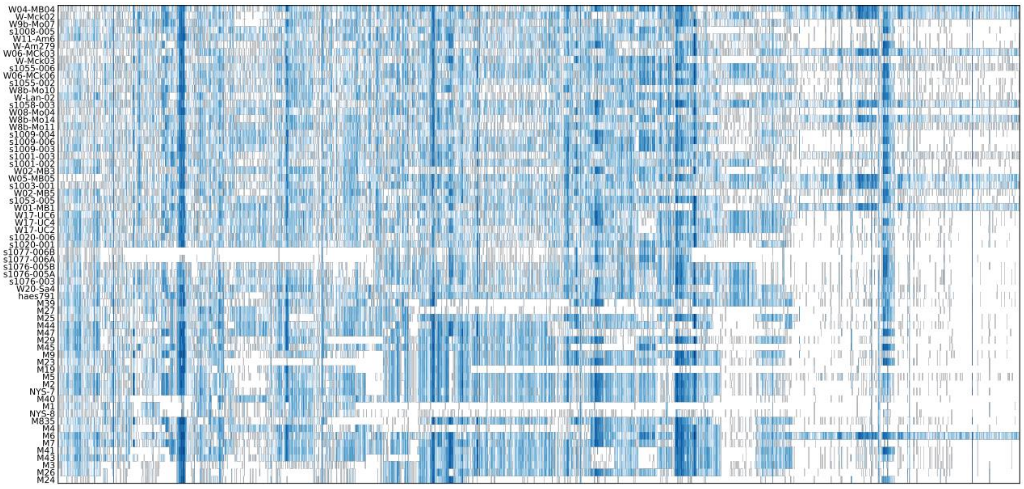

Chr 2

Supplementary Figure 33. Long tracks of homozygosity in Chromosome 2. Levels of heterozygosity were plotted for every 1000bp across 39 wild and 26 varieties accessions with low levels of admixture. A heatmap of heterozygosity is plotted where white indicates no heterozygosity and dark blue indicates high heterozygosity.

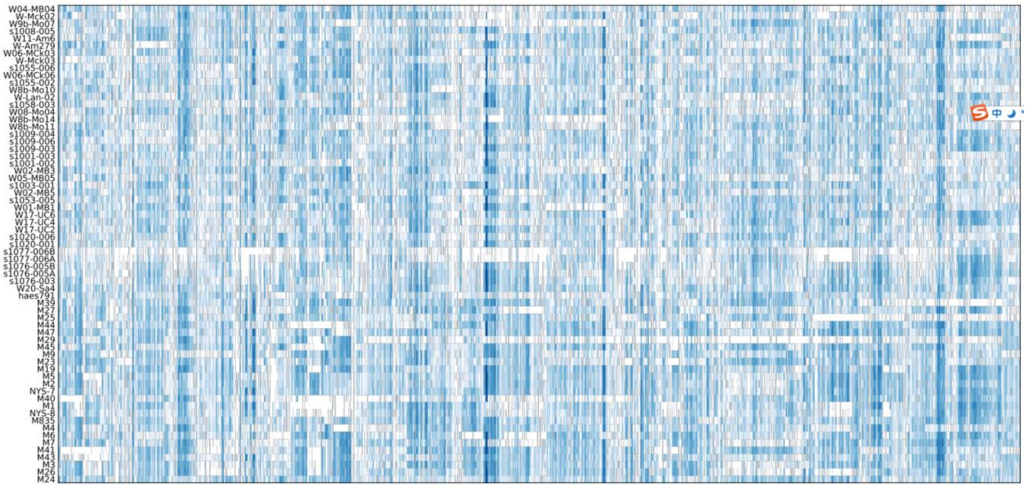

Chr 3

Supplementary Figure 34. Long tracks of homozygosity in Chromosome 3. Levels of heterozygosity were plotted for every 1000bp across 39 wild and 26 varieties accessions with low levels of admixture. A heatmap of heterozygosity is plotted where white indicates no heterozygosity and dark blue indicates high heterozygosity.

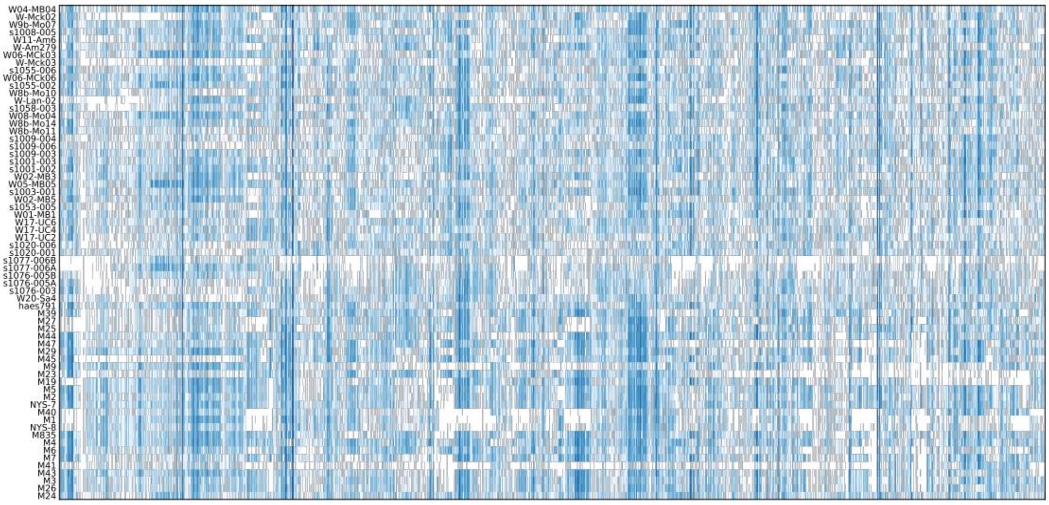

Chr 4

Supplementary Figure 35. Long tracks of homozygosity in Chromosome 4. Levels of heterozygosity were plotted for every 1000bp across 39 wild and 26 varieties accessions with low levels of admixture. A heatmap of heterozygosity is plotted where white indicates no heterozygosity and dark blue indicates high heterozygosity.

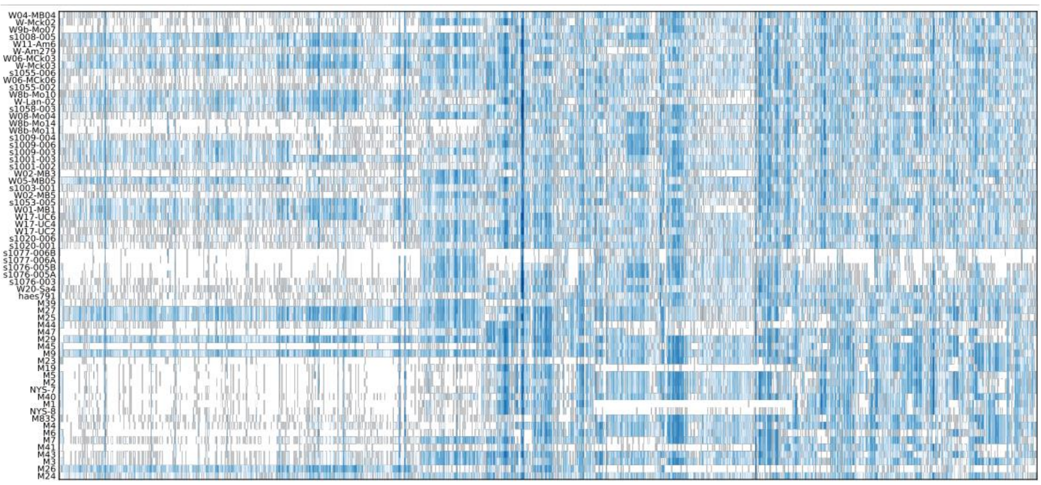

Chr 5

Supplementary Figure 36. Long tracks of homozygosity in Chromosome 5. Levels of heterozygosity were plotted for every 1000bp across 39 wild and 26 varieties accessions with low levels of admixture. A heatmap of heterozygosity is plotted where white indicates no heterozygosity and dark blue indicates high heterozygosity.

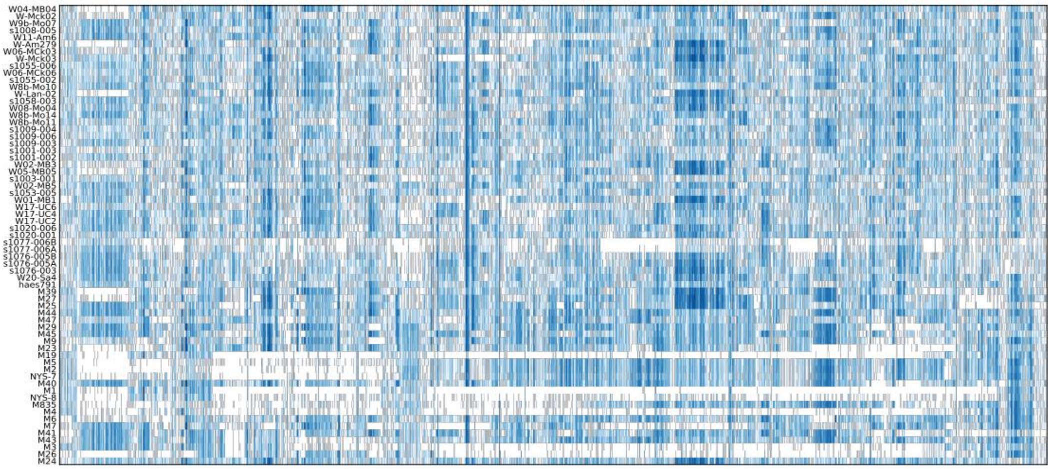

Chr 6

Supplementary Figure 37. Long tracks of homozygosity in Chromosome 6. Levels of heterozygosity were plotted for every 1000bp across 39 wild and 26 varieties accessions with low levels of admixture. A heatmap of heterozygosity is plotted where white indicates no heterozygosity and dark blue indicates high heterozygosity.

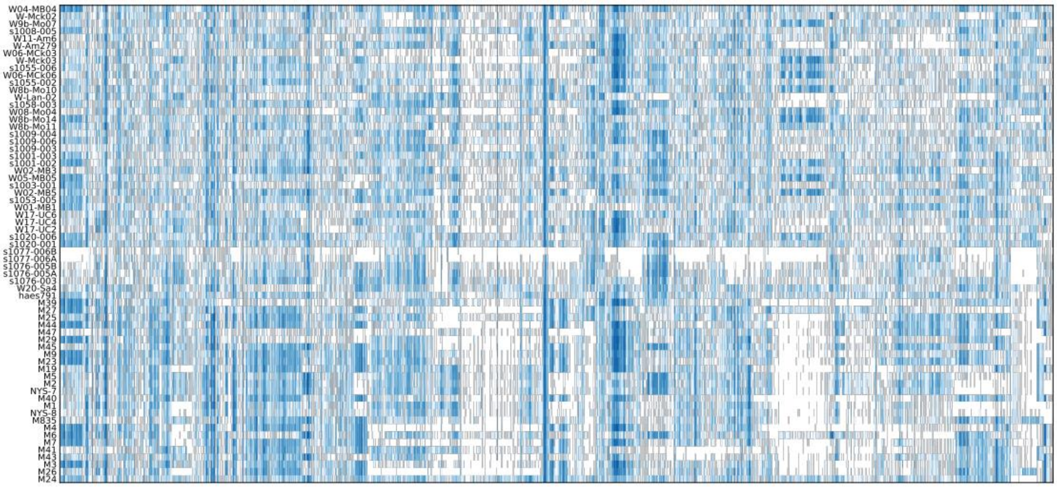

Chr 7

Supplementary Figure 38. Long tracks of homozygosity in Chromosome 7. Levels of heterozygosity were plotted for every 1000bp across 39 wild and 26 varieties accessions with low levels of admixture. A heatmap of heterozygosity is plotted where white indicates no heterozygosity and dark blue indicates high heterozygosity.

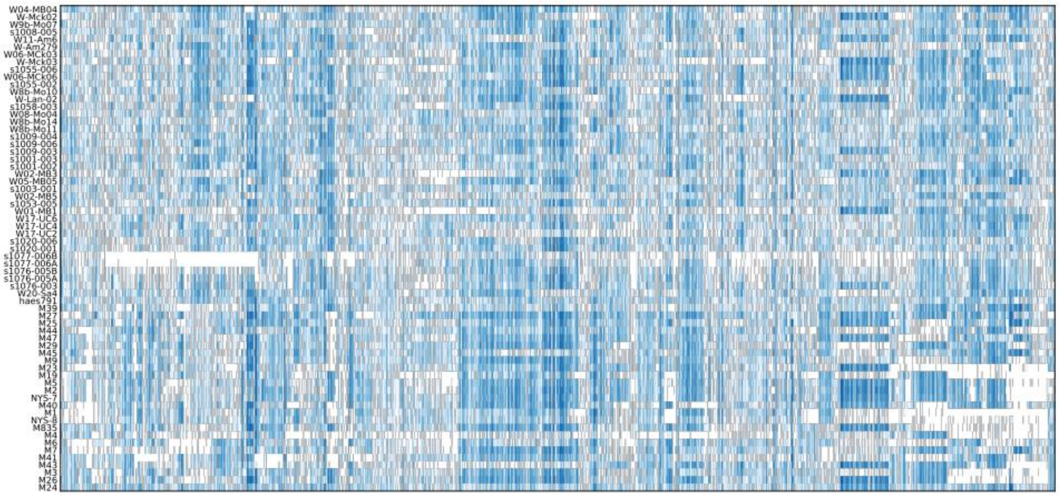

Chr 8

Supplementary Figure 39. Long tracks of homozygosity in Chromosome 8. Levels of heterozygosity were plotted for every 1000bp across 39 wild and 26 varieties accessions with low levels of admixture. A heatmap of heterozygosity is plotted where white indicates no heterozygosity and dark blue indicates high heterozygosity.

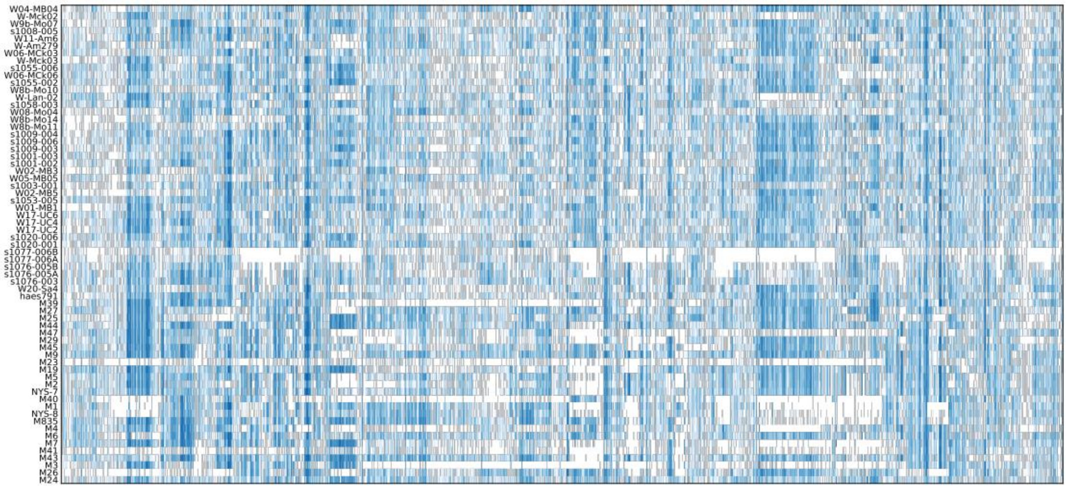

Chr 9

Supplementary Figure 40. Long tracks of homozygosity in Chromosome 9. Levels of heterozygosity were plotted for every 1000bp across 39 wild and 26 varieties accessions with low levels of admixture. A heatmap of heterozygosity is plotted where white indicates no heterozygosity and dark blue indicates high heterozygosity.

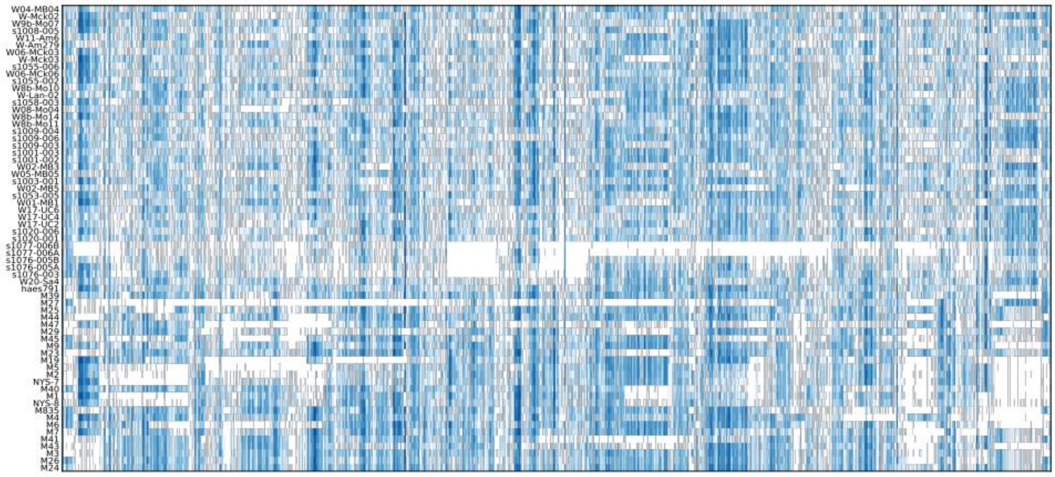

Chr 10

Supplementary Figure 41. Long tracks of homozygosity in Chromosome 10. Levels of heterozygosity were plotted for every 1000bp across 39 wild and 26 varieties accessions with low levels of admixture. A heatmap of heterozygosity is plotted where white indicates no heterozygosity and dark blue indicates high heterozygosity.

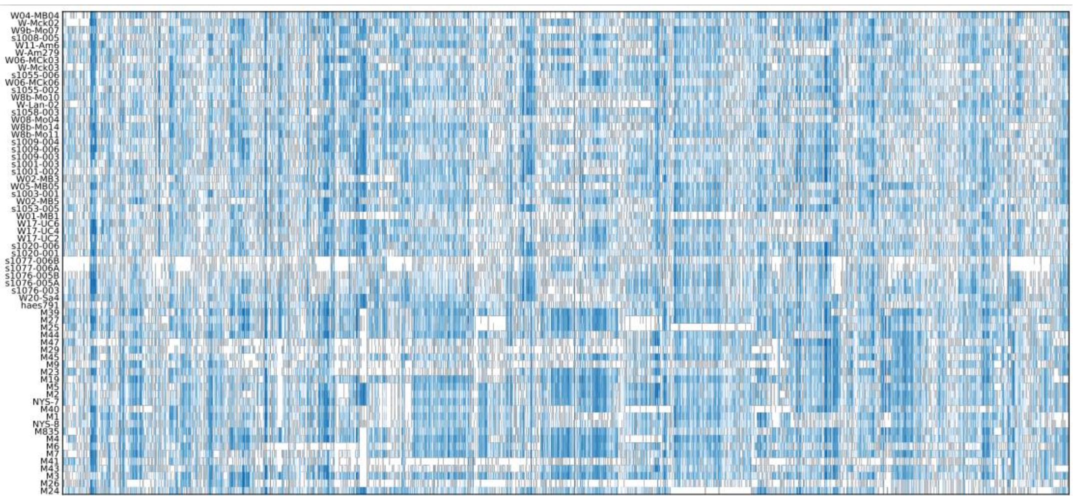

Chr 11

Supplementary Figure 42. Long tracks of homozygosity in Chromosome 11. Levels of heterozygosity were plotted for every 1000bp across 39 wild and 26 varieties accessions with low levels of admixture. A heatmap of heterozygosity is plotted where white indicates no heterozygosity and dark blue indicates high heterozygosity.

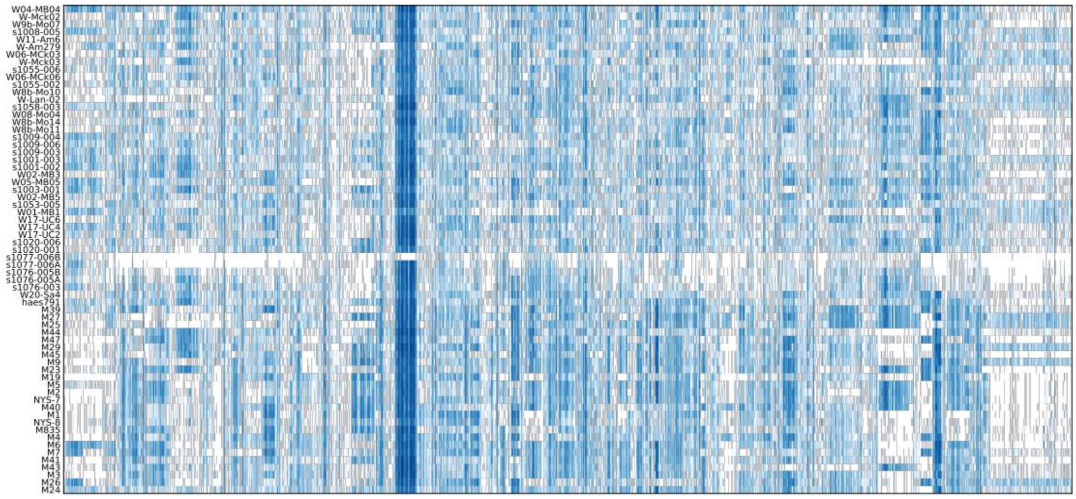

Chr 12

Supplementary Figure 43. Long tracks of homozygosity in Chromosome 12. Levels of heterozygosity were plotted for every 1000bp across 39 wild and 26 varieties accessions with low levels of admixture. A heatmap of heterozygosity is plotted where white indicates no heterozygosity and dark blue indicates high heterozygosity.

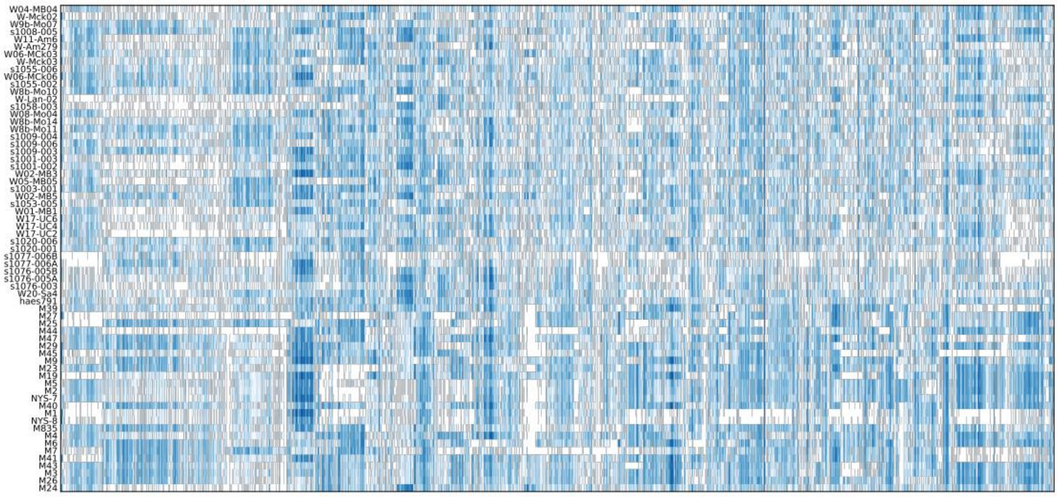

Chr 13

Supplementary Figure 44. Long tracks of homozygosity in Chromosome 13. Levels of heterozygosity were plotted for every 1000bp across 39 wild and 26 varieties accessions with low levels of admixture. A heatmap of heterozygosity is plotted where white indicates no heterozygosity and dark blue indicates high heterozygosity.

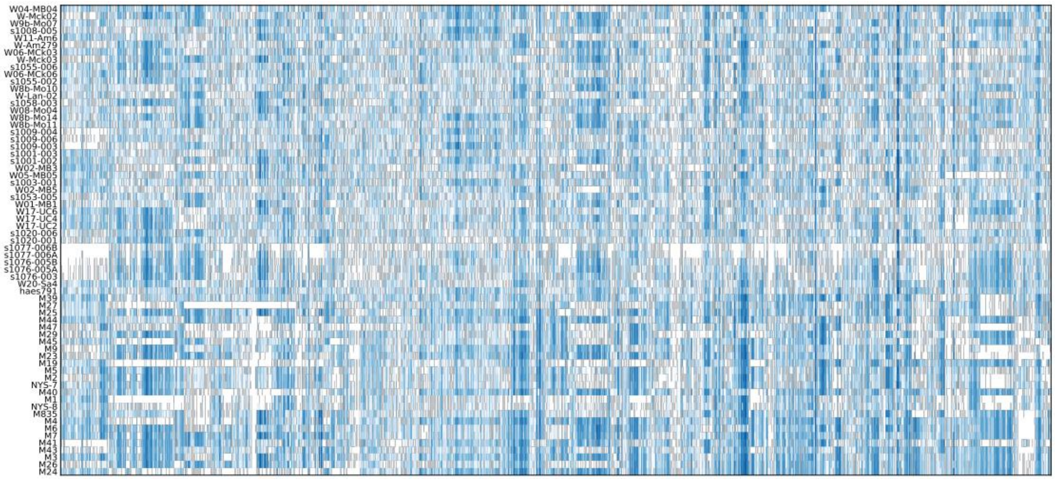

Chr 14

Supplementary Figure 45. Long tracks of homozygosity in Chromosome 14. Levels of heterozygosity were plotted for every 1000bp across 39 wild and 26 varieties accessions with low levels of admixture. A heatmap of heterozygosity is plotted where white indicates no heterozygosity and dark blue indicates high heterozygosity.
